# Progranulin aggravates lethal *Candida albicans* sepsis by regulating inflammatory response and antifungal immunity

**DOI:** 10.1371/journal.ppat.1010873

**Published:** 2022-09-19

**Authors:** Jiayu Liu, Xiaofei Lai, Renlin Yu, Hao Ding, Haobo Bai, Zhubin Yang, Yibing Yin, Fang Xu, Ju Cao

**Affiliations:** 1 Department of Laboratory Medicine, The First Affiliated Hospital of Chongqing Medical University, Chongqing, China; 2 Key Laboratory of Laboratory Medical Diagnostics Designated by the Ministry of Education, School of Laboratory Medicine, Chongqing Medical University, Chongqing, China; 3 Department of Intensive Care Unit, The First Affiliated Hospital of Chongqing Medical University, Chongqing, China; University of Michigan Health System, UNITED STATES

## Abstract

*Candida albicans* is the most frequent pathogen of fungal sepsis associated with substantial mortality in critically ill patients and those who are immunocompromised. Identification of novel immune-based therapeutic targets from a better understanding of its molecular pathogenesis is required. Here, we reported that the production of progranulin (PGRN) levels was significantly increased in mice after invasive *C*.*albicans* infection. Mice that lacked PGRN exhibited attenuated kidney injury and increased survival upon a lethal systemic infection with *C*. *albicans*. In mice, PGRN deficiency protected against systemic candidiasis by decreasing aberrant inflammatory reactions that led to renal immune cell apoptosis and kidney injury, and by enhancing antifungal capacity of macrophages and neutrophils that limited fungal burden in the kidneys. PGRN in hematopoietic cell compartment was important for this effect. Moreover, anti-PGRN antibody treatment limited renal inflammation and fungal burden and prolonged survival after invasive *C*. *albicans* infection. *In vitro*, PGRN loss increased phagocytosis, phagosome formation, reactive oxygen species production, neutrophil extracellular traps release, and killing activity in macrophages or neutrophils. Mechanistic studies demonstrated that PGRN loss up-regulated Dectin-2 expression, and enhanced spleen tyrosine kinase phosphorylation and extracellular signal-regulated kinase activation in macrophages and neutrophils. In summary, we identified PGRN as a critical factor that contributes to the immunopathology of invasive *C*.*albicans* infection, suggesting that targeting PGRN might serve as a novel treatment for fungal infection.

## Author summary

*Candida albicans* is a frequent etiological agent of fungal sepsis associated with high mortality in critically ill patients and those who are immunocompromised. Considering the limited scope of currently available antifungal drugs, adjuvant immunotherapy may have the potential to reduce the burden of mortality caused by *C*.*albicans* sepsis. Therefore, there is an increasingly pressing need to develop novel immune response-based therapies. In this study, we found that progranulin (PGRN) production was increased in mice after invasive *C*.*albicans* infection. PGRN-deficient mice had significantly higher survival rate upon a lethal systemic infection with *C*.*albicans*. In mice, PGRN deficiency protected against systemic candidiasis by dampening inflammation and by limiting fungal burden in the kidney, the primary infected organ in the mouse model of systemic candidiasis. Remarkably, we showed that anti-PGRN antibody treatment limited renal inflammation and fungal burden and prolonged survival after invasive *C*. *albicans* infection. Furthermore, PGRN deficiency enhanced antifungal capacity of macrophages and neutrophils by increasing fungal phagocytosis and killing. Thus, PGRN might be a therapeutic target for treating fungal infection. Our findings shed new light on the negative regulation of inflammatory reaction and immune response during *C*.*albicans* infection, and may have translationally relevant implications for developing novel approaches to treat fungal sepsis.

## Introduction

Invasive fungal infection is a growing health burden, especially in critically ill patients and those who are immunocompromised [1]. *Candida albicans* is the most frequent clinical fungal pathogen, which can invade the mucosa reaching the bloodstream and causing invasive *C*.*albicans* infection (IC) and sepsis [2]. Currently, IC is associated with a crude mortality of ~40–55% in intensive care unit (ICU) [3]. Considering the limited numbers of antifungal drugs and the emergence of drug-resistant *Candida* strains, it is believed that adjuvant immunotherapy will have the capacity to reduce the burden of morbidity and mortality caused by IC.

The host immunity directs the protection against IC in two main ways: clearance of invading fungi and inhibition of fatal infection-related inflammation and damage [4]. C-type lectin receptors (CLRs), such as Dectin-1, Dectin-2, and Mincle, expressed on host immune cells including macrophages and neutrophils, recognize fungal cell wall constituents such as β-glucans, α-mannans, and glycolipids, which initiates the downstream signaling cascades critical for host immunity against IC [5–7]. Dectin-1, Dectin-2 and Mincle all signal via tyrosine-based motifs that recruit the spleen tyrosine kinase (Syk) [8]. Syk activation contributes essentially to reactive oxygen species (ROS) production, phagosomal maturation, phagocytosis, neutrophil extracellular traps (NETs) formation, and elimination of phagocytosed fungal pathogens in macrophages or neutrophils [9–11]. Maintaining balance between the control of fungal burden and the excessive inflammation has important implications for the development of immune-based therapeutics in the treatment of IC.

Progranulin (PGRN) is a pleiotropic modulator critical for immunology by regulating various signaling pathways [12]. PGRN was reported to protect against bacterial pneumonia and sepsis [13–15], but it aggravated pulmonary immunopathology during influenza pneumonia [16,17]. Although the pathophysiology of bacterial sepsis is significantly different from fungal sepsis [18,19], the physiological function of PGRN in response to fungal infection remains unknown. Here we showed an unexpectedly detrimental role of PGRN in *C*.*albicans* sepsis.

## Results

### PGRN expression was up-regulated after C. albicans infection

**Fig 1:**
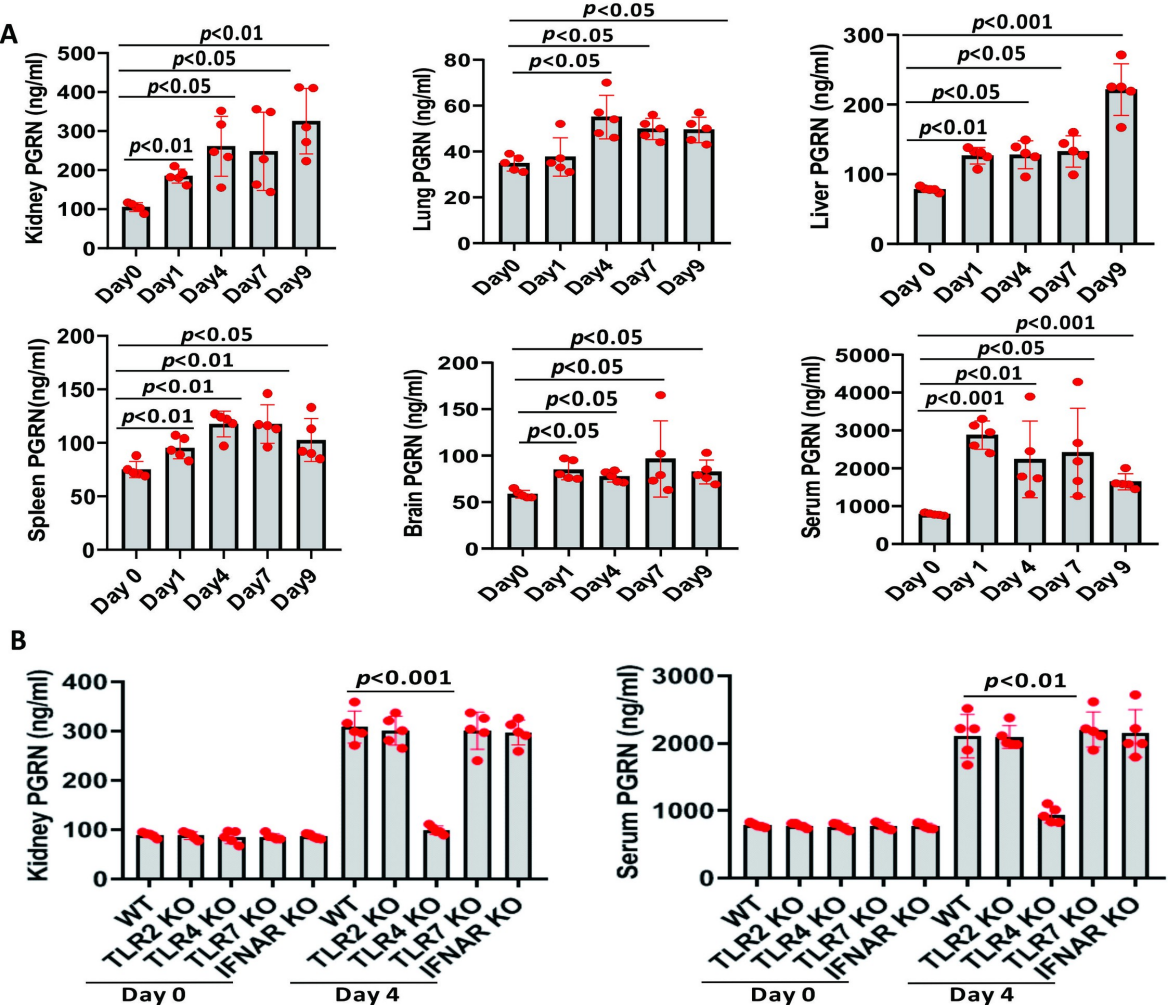
Local and systemic PGRN production in mice after invasive *C*.*albicans* infection. Wild type (WT, n = 5) mice were infected intravenously with 4 × 10^5^ colony forming units (CFU) of *C*. *albicans*. (A) Organs were removed at the indicated time points, and blood was collected by cardiac puncture. Samples were assayed for PGRN content by specific sandwich enzyme-linked immunosorbent assay (ELISA). (B) PGRN concentrations in the kidney and blood from Toll-like receptor (TLR) 2 knockout (KO), TLR4 KO, TLR7 KO, type I IFN-α/β receptor (IFNAR) KO, and WT mice at the indicated times after intravenous infection with 4 x10^5^ CFU of *C*. *albicans*. All data were pooled from three independent experiments. The statistical differences were determined by Kruskal-Wallis test followed by Dunn’s multiple comparisons post test, and *p* values were shown when compared between groups denoted by horizontal lines.

A systemic candidiasis mouse model was established according to previous studies [20,21], in which the kidney was the primary target organ, and mice developed renal failure and sepsis, and this recapitulated the progressive sepsis seen in humans during severe clinical cases. Firstly, we found that PGRN levels in the kidney, lung, liver, spleen, brain and blood were substantially increased at day 1 and lasted to day 9 after invasive *C*. *albicans* infection (Fig 1A). Besides, experiments with Toll-like receptor (TLR)2 knockout (KO), TLR4 KO, TLR7 KO, and type I IFN-α/β receptor (IFNAR) KO mice demonstrated that TLR4 KO mice had significantly lower PGRN levels when compared with wild type (WT) mice in the kidney and blood at day 4 after invasive *C*. *albicans* infection (Fig 1B), suggesting that TLR4 signaling mediated PGRN expression in *C*. *albicans infection*.

### Loss of PGRN ameliorated fungal pathology after IC

**Fig 2:**
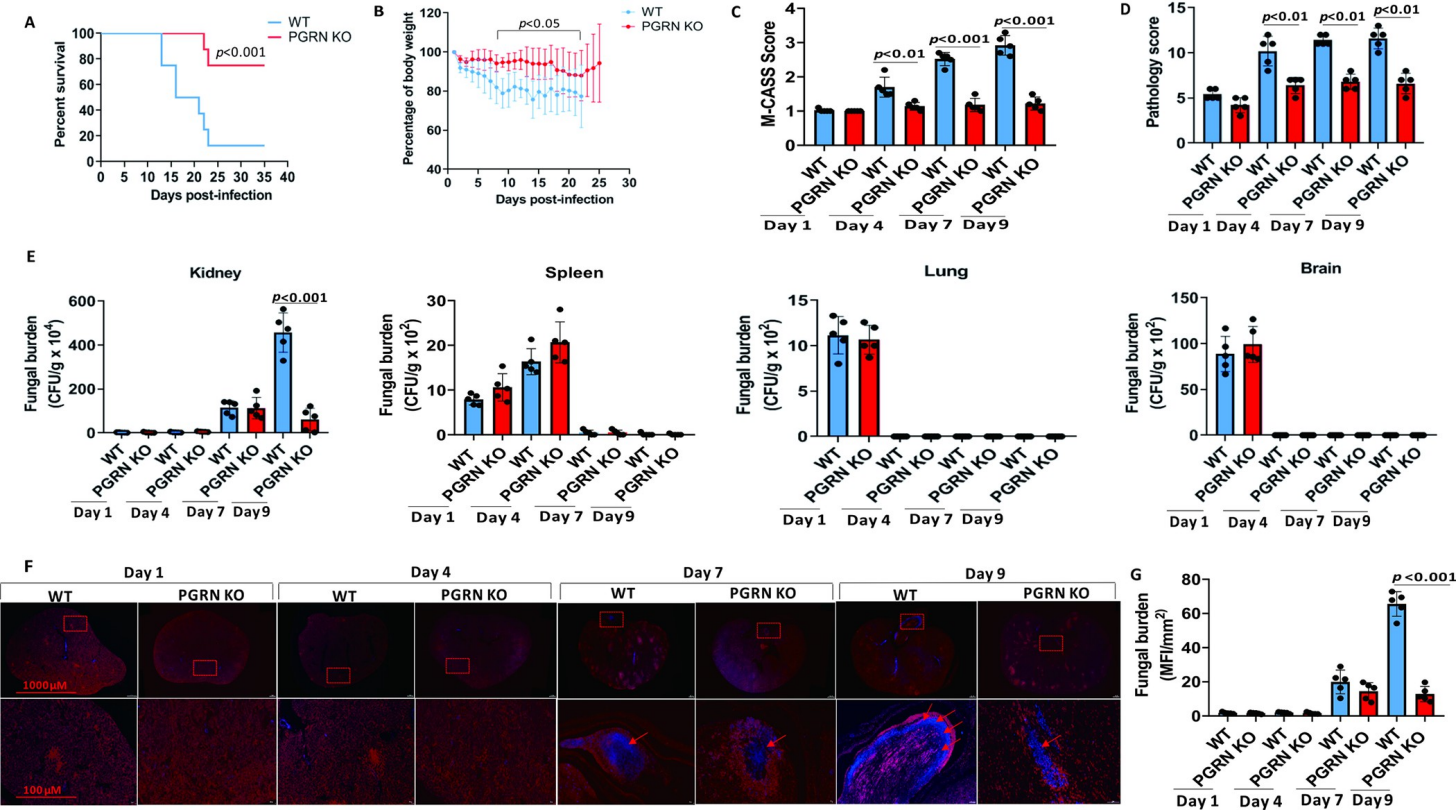
PGRN deletion decreased the susceptibility to invasive *C*.*albicans* infection in mice. (A) PGRN knockout (KO, n = 20) and wild type (WT, n = 20) mice were infected intravenously with 4 × 10^5^ colony forming units (CFU) of *C*. *albicans*. Survival was monitored over indicated time periods. (B) Weight changes of mice after intravenous infection with 4 × 10^5^ CFU of *C*.*albicans* in WT (n = 10) and PGRN KO (n = 10) mice. (C) Mouse Clinical Assessment Score for Sepsis (M-CASS) scores of WT (n = 5) and PGRN KO (n = 5) mice after intravenous infection with 4 × 10^5^ CFU of *C*.*albicans*. (D) Pathology scores in the kidneys after intravenous infection with 4 × 10^5^ CFU of *C*.*albicans* in WT (n = 5) and PGRN KO (n = 5) mice. (E) Fungal titers in the tissues, including kidneys, spleens, lungs and brains, were determined at the indicated times after intravenous infection with 4 × 10^5^ CFU of *C*.*albicans* in WT (n = 5) and PGRN KO (n = 5) mice. (F) Representative Calcofluro White (CW) staining of kidney sections from WT (n = 5) and PGRN KO (n = 5) mice after invasive *C*.*albicans* infection. Red arrows indicated hyphae of blue staining. Bottom panels are high magnification. Scale bars are 100 μM. (G) Fungal load score by analysis of CW-stained kidney sections at the indicated times after invasive *C*.*albican*s infection in WT (n = 5) and PGRN KO (n = 5) mice. MFI = mean fluorescence intensity. All data were pooled from three independent experiments. Log-rank test was used to analyze the difference between survival curves. The Mann–Whitney *U* test was used to analyze the difference of other parameters between WT and PGRN KO mice at the same time point after *C*.*albicans* infection, and *p* values were shown when compared between groups denoted by horizontal lines.

To investigate the role of PGRN in candidiasis, we intravenously infected PGRN KO and WT control mice with a lethal dose of *C*. *albicans*. Strikingly, PGRN deficiency significantly increased the survival of *C*. *albicans*-infected mice compared with WT controls (Fig 2A). Consistently, PGRN KO mice experienced significantly less body weight loss (Fig 2B), and Mouse Clinical Assessment Score for Sepsis (M-CASS) scores were significantly decreased in PGRN KO mice compared with WT mice at days 4, 7 and 9 after *C*. *albicans* challenge (Figs 2C and S1A). The kidneys of PGRN KO mice appeared pinker, less swollen, and had less distinguishable nodules than the kidneys of WT mice at days 4, 7, and 9 after invasive *C*.*albicans* infection (S1B Fig). Histopathological analysis showed that WT kidneys had numerous multifocal areas of abscess formation and manifested increased renal inflammation compared with PGRN KO kidneys at days 4, 7, and 9 after invasive *C*.*albicans* infection (S1C Fig), which was reflected by significantly higher pathology scores in WT mice compared with PGRN KO mice (Fig 2D). In the kidney, the fungal burden was similar in all mice at days 1, 4 and 7, whereas in PGRN KO mice, the fungal burden was significantly lower at day 9 of *C*.*albicans* injection than in WT mice (Fig 2E). Calcofluro White staining also clearly revealed less prominent hyphae within abscesses of PGRN KO versus WT kidneys (Fig 2F), which reached statistical difference at day 9 after *C*.*albicans* injection (Fig 2G). In other organs (spleen, lung and brain), fungal burden was controlled by the immune system and progressively decreased to be undetected over time, and no differences in fungal burden were observed in PGRN KO and WT mice (Fig 2E). Therefore, our data showed that at an early phase (during the first week) of invasive *C*. *albicans* infection, the loss of PGRN did not affect fungal burden in the kidney, whereas, at a later phase of infection (day 9) fungal burden was significantly lower in PGRN-deficient mice than in control WT mice. Anyway, these findings demonstrated that PGRN deficiency protected mice from lethal systemic infection with *C*. *albicans*.

### Hematopoietic-derived PGRN contributed to the enhanced susceptibility of mice to IC

**Fig 3:**
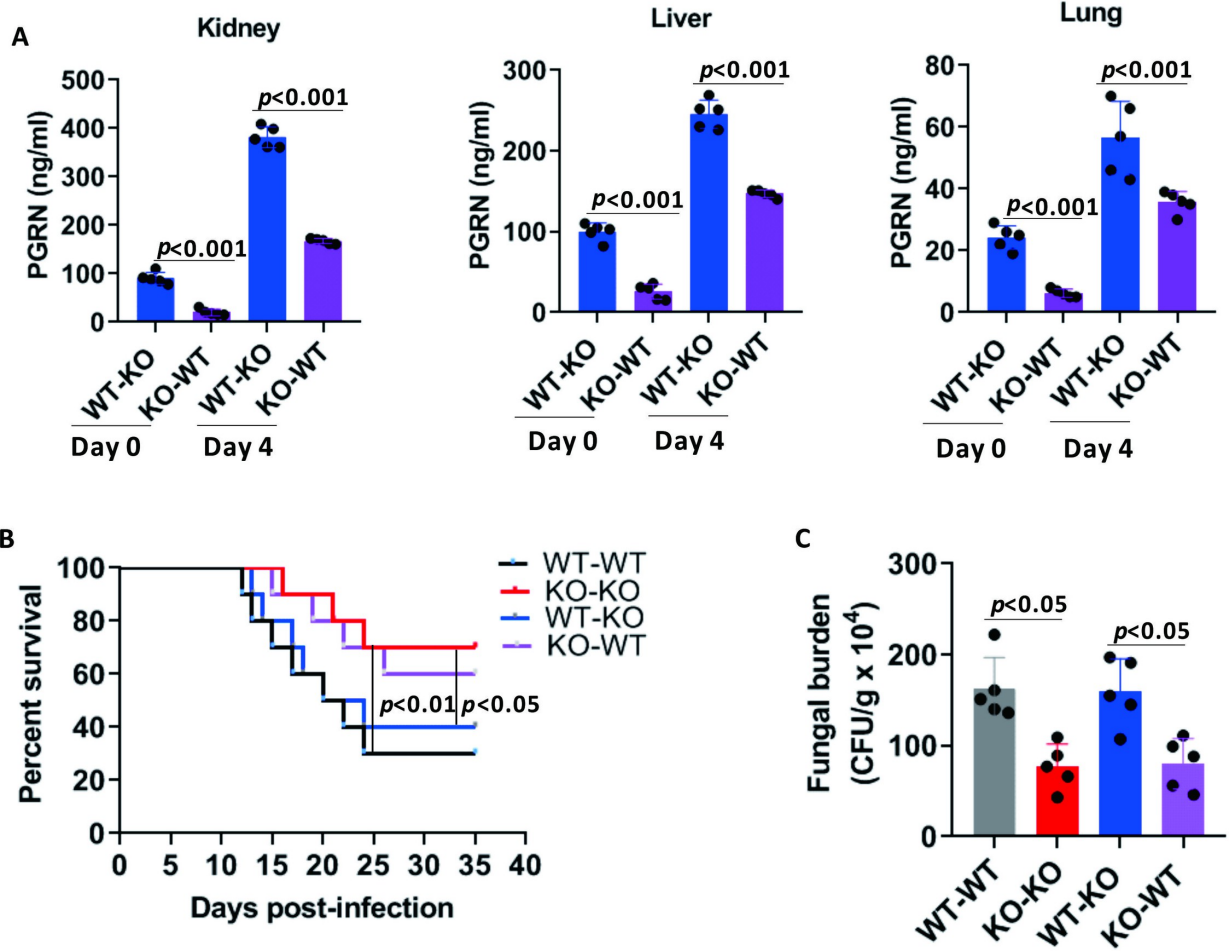
Contribution of PGRN-deficient hematopoietic cells to the increased resistance of PGRN KO mice to invasive *C*.*albicans* infection. (A) Bone-marrow cells from PGRN KO (n = 5) and WT (n = 5) mice were intravenously injected into the irradiated recipient mice separately. Eight weeks later, mice were intravenously infected with 4×10^5^ CFU of *C*. *albica*n*s*, and PGRN levels in the kidney, liver and lung were determined by ELISA. The statistical difference was determined by Kruskal-Wallis test followed by Dunn’s multiple comparisons post test, *p* values were shown when compared between groups denoted by horizontal lines. (B) Bone-marrow cells from PGRN KO (n = 10) and WT (n = 10) mice were intravenously injected into the irradiated recipient mice separately. Eight weeks later, mice were intravenously infected with 4×10^5^ CFU of *C*. *albica*n*s*. Survival of these mice was monitored. Kaplan-Meier survival curves were shown, and *p* value was determined by log-rank survival test. (C) Radiation chimeras were infected with 4×10^5^ CFU of *C*. *albicans* by intravenous inoculation, and kidney CFU levels at day 9 after *C*. *albicans* infection were evaluated. All data were pooled from three independent experiments. The Mann–Whitney *U* test was used to analyze the difference between groups denoted by horizontal lines, and *p* values were shown.

To investigate the cellular basis of the PGRN-related antifungal immunity, we generated bone marrow (BM)-chimeric mice by reconstituting lethally irradiated WT mice with syngeneic PGRN KO BM, or PGRN KO mice with WT BM. PGRN KO mice transplanted with WT BM had significantly increased PGRN concentrations in the kidneys, livers and lungs after *C*. *albicans* infection compared with WT mice transplanted with PGRN KO BM (Fig 3A). Transplantation of PGRN KO BM into irradiated WT or PGRN KO recipients significantly enhanced the survival of *C*. *albicans*-induced sepsis relative to the results seen with WT BM (Fig 3B). Additionally, irradiated WT and PGRN KO mice reconstituted with PGRN KO BM displayed a significant reduction in the fungal burden of kidney relative to mice receiving WT BM at day 9 after infection (Fig 3C). These data therefore suggest that PGRN derived from the hematopoietic cell compartment contributed to decreased resistance against invasive fungal infection by *C*. *albicans*.

### PGRN deficiency reduced the inflammatory host response to IC

**Fig 4:**
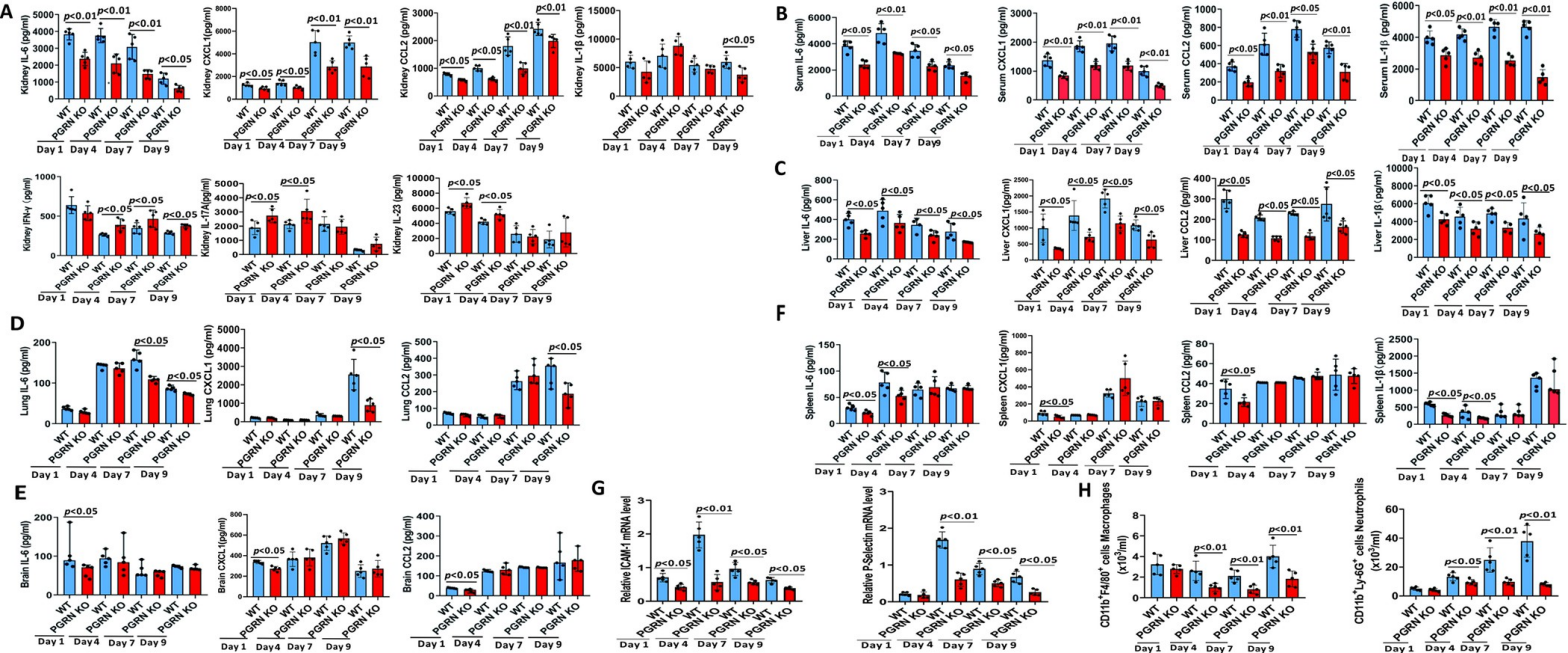
PGRN deletion mediated inflammatory responses in mice after invasive *C*.*albicans* infection. PGRN KO (n = 5) and WT (n = 5) mice were infected intravenously with 4 × 10^5^ colony forming units (CFU) of *C*. *albicans*, and then inflammatory mediators and leukocytes were quantified at the indicated times. (A) Cytokines and chemokines in the kidneys were quantified by enzyme-linked immunosorbent assay (ELISA) at the indicated times after *C*. *albicans* infection. (B) Cytokines and chemokines in the sera were quantified by ELISA at the indicated times after *C*. *albicans* infection. (C) Cytokines and chemokines in the livers were quantified by ELISA at the indicated times after *C*. *albicans* infection. (D) Cytokines and chemokines in the lungs were quantified by ELISA at the indicated times after *C*. *albicans* infection. (E) Cytokines and chemokines in the brains were quantified by ELISA at the indicated times after *C*. *albicans* infection. (F) Cytokines and chemokines in the spleens were quantified by ELISA at the indicated times after *C*. *albicans* infection. (G) Gene expression levels of ICAM-1 and P-Selectin in the kidneys were quantified by quantitative PCR at the indicated times after *C*. *albicans* infection. (H) Kidney cells from *C*. *albicans*-infected WT and PGRN KO mice were stained with anti-Ly-6G and anti-F4/80 antibodies, and FACS plots in S3 Fig showed the gating strategy for macrophages (CD11b^+^F4/80^+^) and neutrophils (CD11b^+^Ly-6G^+^). Graphs showed the absolute numbers of macrophages and neutrophils in homogenized kidneys. All data were pooled from three independent experiments. The Mann–Whitney *U* test was used to analyze the difference between WT and PGRN KO mice at the same time point after *C*.*albicans* infection, and *p* values were shown when compared between groups denoted by horizontal lines.

Aberrant host inflammatory response has been reported to contribute essentially to death in IC [19,22]. To address the possibility that the increased survival of PGRN-deficient mice to IC could be due to the effects on inflammatory response, we quantified the levels of proinflammatory cytokines and chemokines after *C*. *albicans* infection. In agreement with reduced renal inflammation in PGRN KO mice by histopathological analysis (S1C Fig), at early time points of infection (days 1, 4 and 7) when similar fungal burden was observed in WT and PGRN KO mice (Fig 2E–2G), we found that kidney IL-6, CXCL1 and CCL2 levels in PGRN KO mice were significantly lower than those in WT mice (Fig 4A). In addition to IL-6, CXCL1 and CCL2, IL-1β protein levels in PGRN KO kidney were also significantly lower than WT mice at day 9 post-infection when significantly lower fungal burden was observed in PGRN KO mice (Fig 2E–2G). Interestingly, the renal levels of IFN-γ (at days 4, 7 and 9), IL-17A (at days 1 and 4) and IL-23 (at days 1 and 4) were significantly higher in PGRN KO mice than those in WT mice after *C*. *albicans* infection. However, there was no significant difference in the production of renal TNF-α, IL-12, GM-CSF, IL-18, IL-2, IL-10 and IL-15 in PGRN KO mice compared with WT mice (S2A Fig). Furthermore, we found that the serum levels of IL-6, CXCL1, CCL2 and IL-1β in PGRN KO mice were significantly lower than those in WT mice at days 1, 4, 7 and 9 after *C*. *albicans* infection (Figs 4B and S2B). In the liver, PGRN KO mice had significantly reduced levels of IL-6, CXCL1, CCL2 and IL-1β at days 1, 4, 7 and 9 after *C*. *albicans* infection compared with WT mice (Figs 4C and S2C). In the lung, PGRN KO mice had significantly lower levels of IL-6 (at days 7 and 9), CXCL1 (at day 9) and CCL2 (at day 9) after *C*. *albicans* infection (Figs 4D and S2D). In the brain, PGRN KO mice displayed significantly lower production of IL-6, CXCL1 and CCL2 at day 1 after *C*. *albicans* infection (Figs 4E and S2E). In the spleen, PGRN KO mice had significantly lower IL-6 (at days 1 and 4), CXCL1 (at day 1), CCL2 (at day 1) and IL-1β (at days 1 and 4) after *C*. *albicans* infection (Figs 4F and S2F). Taken together, these results demonstrated that PGRN modulated the release of proinflammatory cytokines and chemokines, especially in the kidney, which is the primary infected organ, after *C*. *albicans* infection.

We further determined the expression levels of additional inflammation mediators involved in acute kidney inflammation, including the major adhesion molecules: intercellular adhesion molecule 1 (ICAM-1) and P-Selectin, and we detected significantly reduced renal expression levels of ICAM-1 (at days 1, 4, 7, and 9) and P-Selectin (at days 4, 7, and 9) in PGRN KO mice compared with WT mice after *C*. *albicans* infection (Fig 4G). Besides, we analysed, by flow cytometry (S3 Fig), different leucocyte cell types infiltrating the infected kidney of PGRN KO and WT mice after invasive *C*. *albicans* infection. In line with the significantly reduced levels of early neutrophils chemokine CXCL1 and monocyte/macrophage chemokine CCL2 in the kidney (Fig 4A), PGRN KO mice displayed significantly lower numbers of infiltrating F4/80^+^ macrophages and Ly-6G^+^ neutrophils compared with WT mice at days 4, 7 and 9 after *C*. *albicans* infection (Fig 4H). In summary, these results indicated that PGRN expression enhanced the expression of inflammatory mediators, as well as the recruitment of inflammatory leukocytes, which could exacerbate inflammatory responses and might promote fatal immunopathology after invasive *C*. *albicans* infection.

### PGRN deficiency resulted in attenuated kidney injury after IC

**Fig 5:**
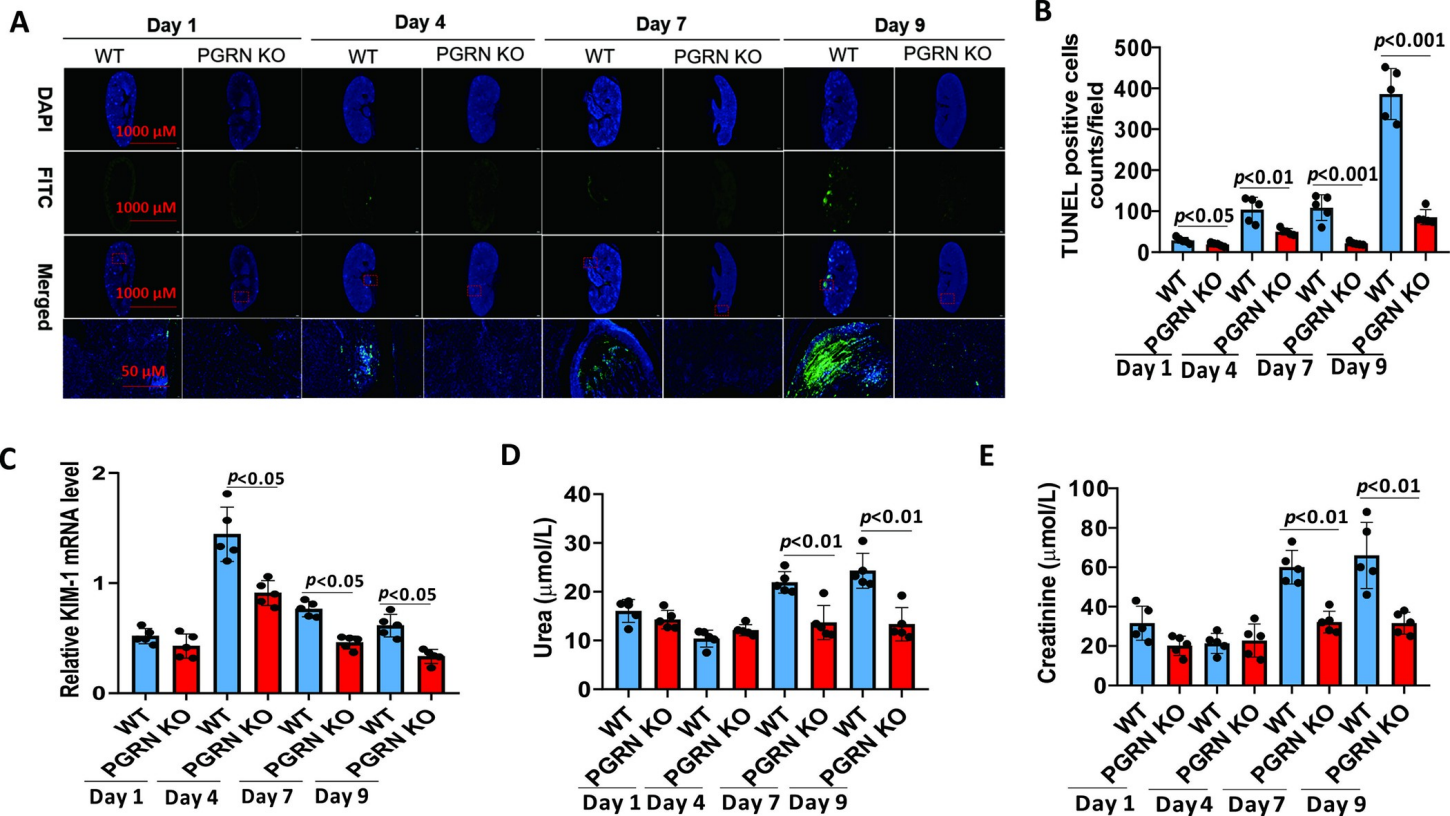
PGRN deletion reduced immune cell apoptosis and tissue injury in the kidneys after invasive *C*.*albicans* infection. PGRN KO (n = 5) and WT (n = 5) mice were infected intravenously with 4 × 10^5^ colony forming units (CFU) of *C*. *albicans*, and then immune cell apoptosis in the kidney and tissue injury markers were assessed at the indicated times. (A) The kidneys from WT (n = 5) and PGRN KO (n = 5) mice at the indicated times after intravenous infection with 4 × 10^5^ CFU of *C*. *albicans* were subjected to DNA fragmentation analysis (terminal deoxynucleotidyltransferase dUTP nick end labeling [TUNEL]). Representative examples were shown. (B) TUNEL-positive cells were counted (n = 5 per group) at the indicated times after intravenous infection with 4 × 10^5^ CFU of *C*. *albicans*. (C) Kidney total RNA was analysed for gene expression of kidney injury marker-1 (KIM-1) at the indicated times after intravenous infection with 4 × 10^5^ CFU of *C*. *albicans*. (D) Serum urea levels in WT (n = 5) and PGRN KO (n = 5) mice after intravenous infection with 4 × 10^5^ CFU of *C*. *albicans*. (E) Serum creatinine levels in WT (n = 5) and PGRN KO (n = 5) mice after intravenous infection with 4 × 10^5^ CFU of *C*. *albicans*. All data were pooled from three independent experiments. The Mann–Whitney *U* test was used to analyze the difference between WT and PGRN KO mice at the same time point after *C*.*albicans* infection, and *p* values were shown when compared between groups denoted by horizontal lines.

To investigate the pathological consequences of the PGRN-mediated inflammatory response and antifungal immunity, we examined kidney functions in PGRN KO and WT mice after IC. Immune cell apoptosis is accompanied by fungal infection and sepsis [23]. Terminal deoxynucleotidyltransferase dUTP nick end labeling (TUNEL) histology analysis showed that PGRN deficiency strongly decreased cell apoptosis in the kidneys after *C*. *albicans* infection compared with WT mice (Fig 5A and 5B). To further assess the kidney failure, we measured the expression level of kidney injury molecule-1 (KIM-1) in the kidneys, which is a marker of early kidney injury, as well as urea and creatinine levels in the sera of infected animals. Compared with WT mice, PGRN KO mice exhibited attenuated kidney injury with a significant reduction in the expression of renal KIM-1 at days 4, 7 and 9 after infection (Fig 5C), as well as significantly lower serum levels of urea (Fig 5D) and creatinine (Fig 5E) at days 7 and 9 after infection. Hence, these data showed that PGRN deficiency led to reduced kidney failure in mice after invasive *C*. *albicans* infection.

### Antibody-mediated inhibition of PGRN ameliorated C.albicans infection

**Fig 6:**
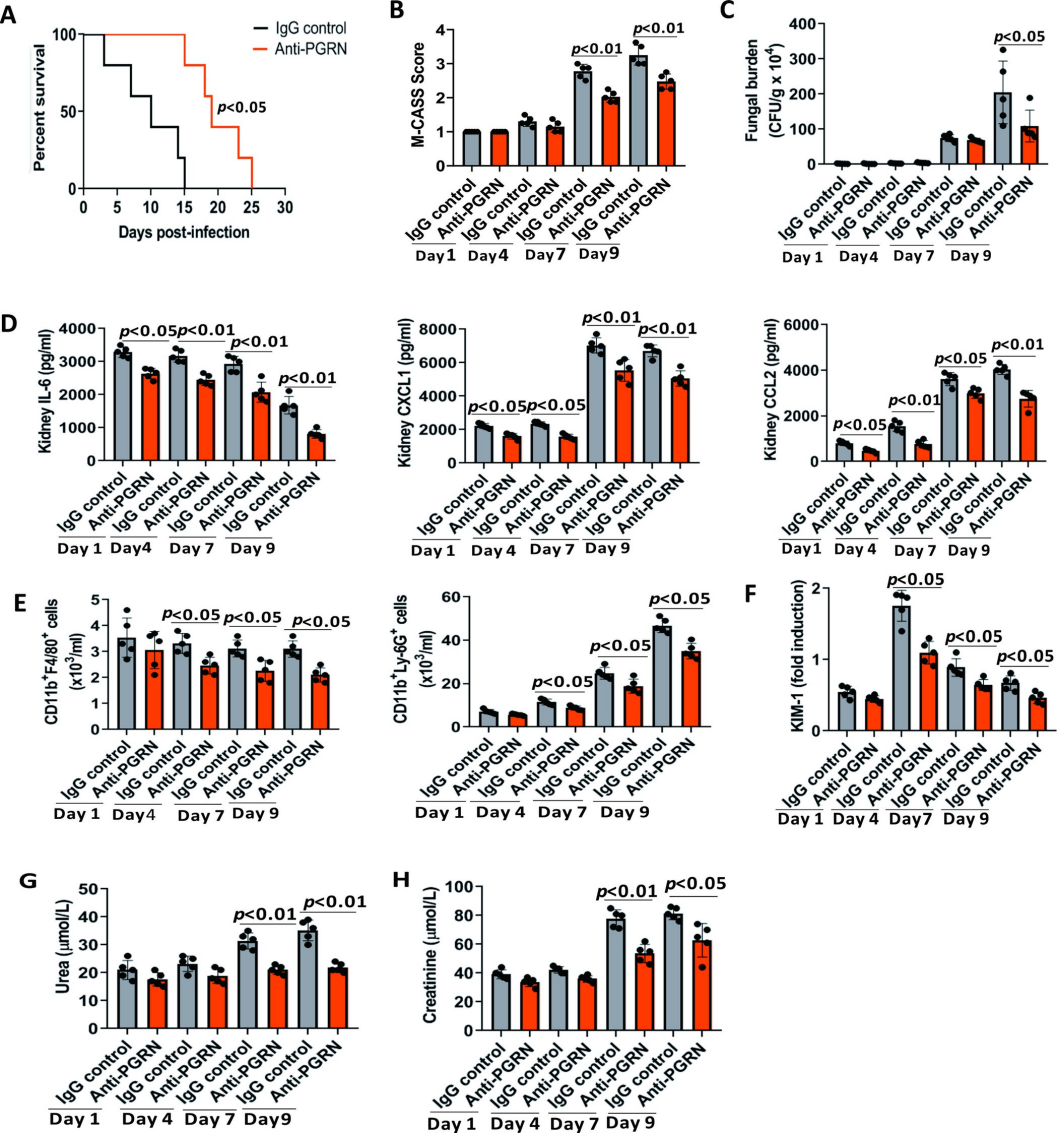
PGRN blockade by anti-PGRN antibody showed therapeutic effect on invasive *C*.*albicans* infection. WT mice were intravenously injected with 1×10^6^ CFU of *C*. *albicans*, and PGRN blockade was performed by intraperitoneally administration of 5 μg of anti-PGRN monoclonal antibody on day 0 (same day as *C*. *albicans* infection), followed by a booster dose of 2.5 μg 24 hour later. (A) Survival of these mice (n = 10 for each group) was monitored. (B) Mouse Clinical Assessment Score for Sepsis (M-CASS) scores of WT mice (n = 5) treated with or without anti-PGRN antibody after intravenous infection with 1 × 10^6^ CFU of *C*.*albicans*. (C) Kidney fungal loading was performed in WT mice (n = 5) treated with or without anti-PGRN antibody at the indicated times after intravenous infection with 1 × 10^6^ CFU of *C*.*albicans*. (D) Kidney IL-6, CXCL1 and CCL2 levels were quantified by ELISA in WT mice (n = 5) treated with or without anti-PGRN antibody at the indicated times after intravenous infection with 1 × 10^6^ CFU of *C*.*albicans*. (E) The numbers of macrophages (CD11b^+^F4/80^+^) and neutrophils (CD11b^+^Ly-6G^+^) in the kidneys were quantified by flow cytometry analysis in WT mice (n = 5) treated with or without anti-PGRN antibody at the indicated times after intravenous infection with 1 × 10^6^ CFU of *C*.*albicans*. (F) The gene expression levels of kidney injury marker-1 (KIM-1) were determined by quantitative PCR in WT mice (n = 5) treated with or without anti-PGRN antibody at the indicated times after intravenous infection with 1 × 10^6^ CFU of *C*.*albicans*. (G) Serum urea levels were measured in WT mice (n = 5) treated with or without anti-PGRN antibody at the indicated times after intravenous infection with 1 × 10^6^ CFU of *C*.*albicans*. (H) Serum creatinine levels were measured in WT mice (n = 5) treated with or without anti-PGRN antibody at the indicated times after intravenous infection with 1 × 10^6^ CFU of *C*.*albicans*. All data were pooled from three independent experiments. Log-rank test was used to analyze the difference between survival curves. The Mann–Whitney *U* test was used to analyze the difference of other parameters between anti-PGRN-treated and control IgG-treated mice at the same time point after *C*.*albicans* infection, and *p* values were shown when compared between groups denoted by horizontal lines.

To further confirm the detrimental role of PGRN in the pathogenesis of lethal *C*. *albicans* sepsis, we took advantage of an anti-PGRN monoclonal antibody that could neutralize PGRN [13]. Administration with an anti-PGRN antibody led to an improved survival time as compared with control IgG upon lethal *C*.*albicans* infection (Fig 6A), and M-CASS scores were significantly lower in mice treated with anti-PGRN antibody at days 7 and 9 after lethal *C*.*albicans* infection (Fig 6B). Quantitatively, fungal titers were significantly lower in the kidneys of anti-PGRN-treated mice at day 9 after infection (Fig 6C). Anti-PGRN treatment also led to significantly reduced protein levels of IL-6, CXCL1, and CCL2 in the kidneys at days 1, 4, 7, and 9 after infection (Fig 6D), and these animals had significantly lower numbers of infiltrating F4/80^+^ macrophages and Ly-6G^+^ neutrophils compared with mice treated with control IgG at days 4, 7 and 9 after *C*. *albicans* infection (Fig 6E). Moreover, anti-PGRN-treated mice exhibited attenuated kidney injury with a significant reduction in the expression of KIM-1 at days 4, 7 and 9 after *C*. *albicans* infection (Fig 6F), as well as significantly lower serum concentrations of urea (Fig 6G) and creatinine (Fig 6H) at days 7 and 9 after infection. Therefore, PGRN blockade might protect mice from *C*. *albicans* sepsis.

### PGRN negatively regulated antifungal activity of macrophages and neutrophils

**Fig 7:**
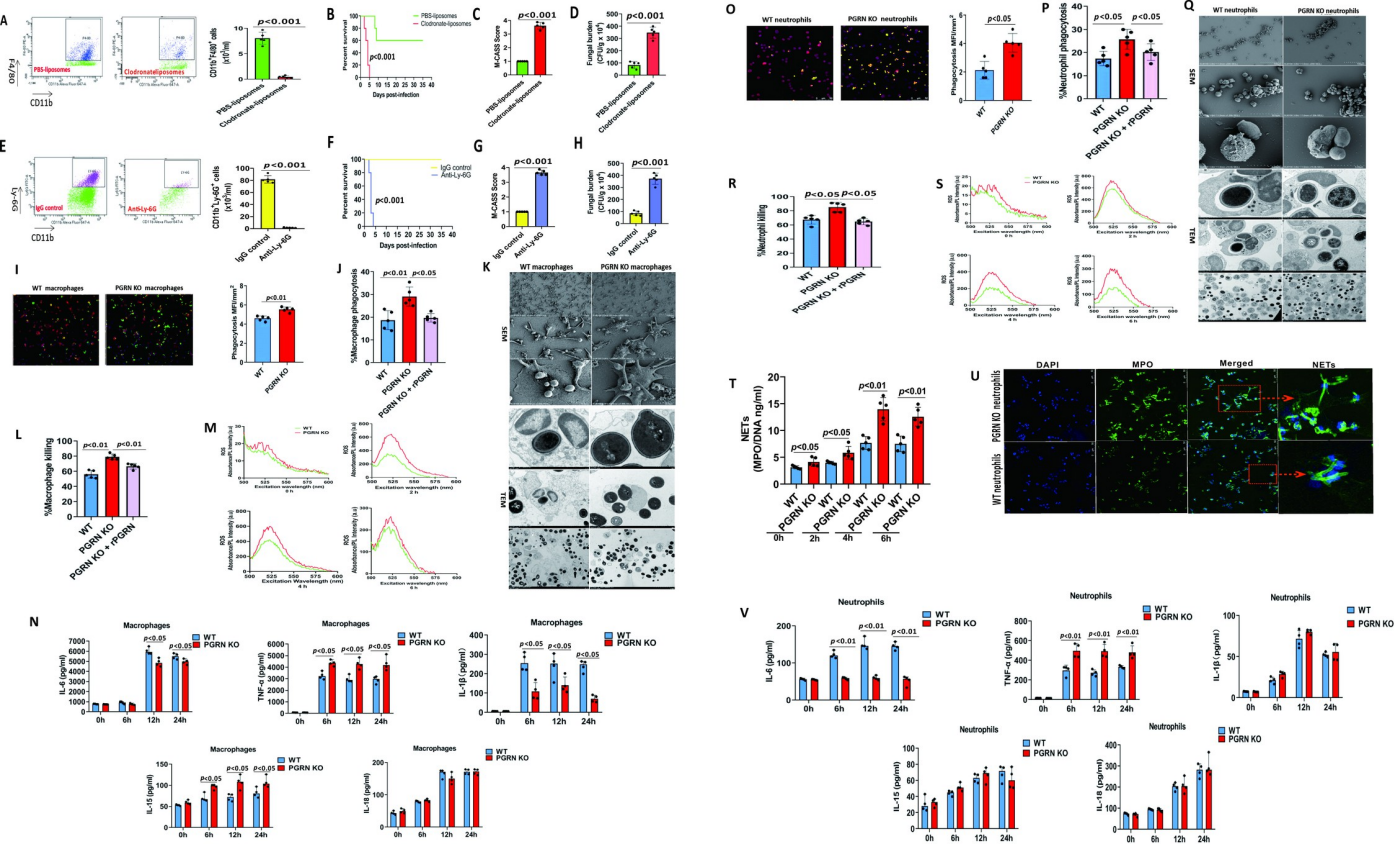
PGRN deletion increased phagocytosis and killing of macrophages and neutrophils in response to fungal infection. (A) The numbers of macrophages (CD11b^+^F4/80^+^) in the spleens were quantified by flow cytometry analysis in PGRN KO mice (n = 5) at 24 hours after clodronate-liposomes treatment. The Mann–Whitney *U* test was used to analyze the difference between groups denoted by horizontal lines. (B) Mortality after macrophage depletion by clodronate-liposomes and subsequent intravenous challenge with 4 x10^5^ CFU of *C*. *albicans* in PGRN KO mice (n = 10). Kaplan-Meier survival curves were shown, and *p* value was determined by log-rank survival test. (C) Mouse Clinical Assessment Score for Sepsis (M-CASS) scores of PGRN KO mice (n = 5) with or without macrophage depletion at day 2 after intravenous infection with 4 × 10^5^ CFU of *C*.*albicans*. The Mann–Whitney *U* test was used to analyze the difference between groups denoted by horizontal lines. (D) Fungal numbers in the kidneys of PGRN KO mice (n = 5) with or without macrophage depletion at day 2 after intravenous infection with 4 × 10^5^ CFU of *C*.*albicans*. The Mann–Whitney *U* test was used to analyze the difference between groups denoted by horizontal lines. (E) The numbers of neutrophils (CD11b^+^Ly-6G^+^) in the spleens were quantified by flow cytometry analysis in PGRN KO mice (n = 5) at 24 hours after anti-Ly-6G antibody treatment. The Mann–Whitney *U* test was used to analyze the difference between groups denoted by horizontal lines. (F) Mortality after neutrophil depletion by anti-Ly-6G antibody and subsequent intravenous challenge with 4 x10^5^ CFU of *C*. *albicans* in PGRN KO mice (n = 10). Kaplan-Meier survival curves were shown, and *p* value was determined by log-rank survival test. (G) M-CASS scores of PGRN KO mice (n = 5) with or without neutrophil depletion at day 2 after intravenous infection with 4 × 10^5^ CFU of *C*.*albicans*. (H) Fungal numbers in the kidneys from PGRN KO mice (n = 5) with or without neutrophil depletion at day 2 after intravenous infection with 4 × 10^5^ CFU of *C*.*albicans*. The Mann–Whitney *U* test was used to analyze the difference between groups denoted by horizontal lines. (I) Rates of phagocytosis for FITC-labeled heat-killed *C*. *albicans* yeast at a multiplicity infection of 1:2 by WT (n = 5) and PGRN-deficient bone marrow-derived macrophages (BMDM, n = 5). Representative fluorescence images of heat-killed *C*. *albicans* yeast (as determined by overlay of green fungus) by WT and PGRN-deficient BMDM were shown. The Mann–Whitney *U* test was used to analyze the difference between groups denoted by horizontal lines. MFI = mean fluorescence intensity. (J) Rates of phagocytosis for live *C*. *albicans* yeast at a multiplicity infection of 1:2 by WT (n = 5) and PGRN-deficient BMDM (n = 5) preincubated with or without recombinant murine PGRN protein (100 ng/ml). The Mann–Whitney *U* test was used to analyze the difference between groups denoted by horizontal lines. (K) WT (n = 5) and PGRN-deficient BMDM (n = 5) were plated on bacteriologic plastic for transmission electron microscopy (TEM) or on coverslips for scanning electron microscopy (SEM) in the same well. BMDM were challenged with heat-killed *C*. *albicans* yeast (at a multiplicity of infection of 1:20) for 30 minutes at 37°C. SEM showed that heat-killed *C*. *albicans* yeasts were no longer seen on the cell surface of PGRN-deficient BMDM, because they had been internalized by the cells (TEM data). In WT BMDM, many incomplete phagocytic cups were observed on the surface of BMDM, showing the arrest of the phagocytic cup closure. Moreover, TEM showed that few heat-killed *C*. *albicans* were internalized in WT BMDM compared with PGRN-deficient BMDM. (L) Percent killing of live *C*. *albicans* by WT (n = 5) and PGRN-deficient BMDM (n = 5) at a multiplicity infection of 1:10 in the presence or absence of recombinant murine PGRN protein (100 ng/ml). The Kruskal-Wallis test followed by Dunn’s multiple comparisons post test was used to analyze the difference between groups denoted by horizontal lines. (M) Reactive oxygen species (ROS) production by WT (n = 5) and PGRN-deficient BMDM (n = 5) stimulated with heat-killed *C*. *albicans* yeast (at a multiplicity of infection of 1:20). Representative examples were shown for the production of ROS. (N) Cytokine and chemokine levels in PGRN KO (n = 5) and WT (n = 5) BMDM at 24 hours after stimulation with heat-killed *C*. *albicans* yeast (at a multiplicity of infection of 1:20). The Mann–Whitney *U* test was used to analyze the difference between groups denoted by horizontal lines. (O) Rates of phagocytosis for FITC-labeled heat-killed *C*. *albicans* yeast at a multiplicity infection of 1:2 by WT (n = 5) and PGRN-deficient (n = 5) neutrophils. Representative fluorescence images of heat-killed *C*. *albicans* (as determined by overlay of yellow fungus) by WT and PGRN-deficient neutrophils were shown. The Mann–Whitney *U* test was used to analyze the difference between groups denoted by horizontal lines. MFI = mean fluorescence intensity. (P) Rates of phagocytosis for live *C*. *albicans* yeast at a multiplicity infection of 1:2 by WT (n = 5) and PGRN-deficient (n = 5) neutrophils preincubated with or without recombinant murine PGRN protein (100 ng/ml). The Mann–Whitney *U* test was used to analyze the difference between groups denoted by horizontal lines. (Q) WT (n = 5) and PGRN-deficient (n = 5) neutrophils were plated on bacteriologic plastic for TEM or on coverslips for SEM in the same well. Bone marrow neutrophils were challenged with heat-killed *C*. *albicans* yeast (at a multiplicity of infection of 1:20) for 30 minutes at 37°C. SEM showed that heat-killed *C*. *albicans* yeasts were no longer seen on the cell surface of PGRN-deficient neutrophils, because they had been internalized by the cells (TEM data). (R) Percent killing of live *C*. *albicans* by WT (n = 5) and PGRN-deficient (n = 5) neutrophils at a multiplicity infection of 1:10 in the presence or absence of recombinant murine PGRN protein (100 ng/ml). The Mann–Whitney *U* test was used to analyze the difference between groups denoted by horizontal lines. (S) ROS production by WT (n = 5) and PGRN-deficient (n = 5) neutrophils stimulated with heat-killed *C*. *albicans* yeast (at a multiplicity of infection of 1:20). Representative examples were shown for the production of ROS. (T) Neutrophils from WT (n = 5) and PGRN-deficient (n = 5) mice were challenged with heat-killed *C*. *albicans* yeast (at a multiplicity of infection of 1:10), and the concentrations of myeloperoxidase (MPO)/DNA-neutrophil extracellular traps (NETs) in the neutrophil culture supernatants were determined at the indicated times after stimulation. The Mann–Whitney *U* test was used to analyze the difference between groups denoted by horizontal lines. (U) Representative fluorescence images of NETs stained for DNA (DAPI, blue) and myeloperoxidase (MPO, green) at 4 hour after *C*. *albicans* stimulation were shown. (V) Cytokine and chemokine levels in PGRN KO (n = 5) and WT (n = 5) neutrophils at 24 hours after stimulation with heat-killed *C*. *albicans* yeast (at a multiplicity of infection of 1:20). The Mann–Whitney *U* test was used to analyze the difference between groups denoted by horizontal lines.

Because macrophages and neutrophils have been reported to be important for the antifungal immunity by phagocytosis and killing mechanisms [4,6,19,24], we thus hypothesized that macrophages and/or neutrophils might mediate the detrimental effect of PGRN on fungal clearance at a later time (day 9, Figs 2EE and 6C) after *C*.*albicans* infection. As expected, macrophage depletion dramatically decreased the survival (Fig 7A and 7B), and significantly increased M-CASS scores (Fig 7C) and impaired renal fungal clearance (Fig 7D) in PGRN KO mice after *C*.*albicans* infection. Meanwhile, neutrophil depletion also greatly decreased the survival of PGRN KO mice (Fig 7E and 7F), and in the absence of neutrophils, M-CASS scores of PGRN KO mice and fungal burden in PGRN KO kidneys were significantly increased after *C*.*albicans* infection (Fig 7G and 7H). These results suggest that both macrophages and neutrophils mediated the beneficial effects of PGRN loss upon *C*.*albicans* sepsis.

We then performed an *ex vivo* phagocytosis and killing assay by co-culturing *C*. *albicans* with bone marrow-derived macrophages (BMDM) or neutrophils, and found that PGRN-deficient macrophages had a significantly higher capacity to phagocytose fluorescein isothiocyanate (FITC)-labeled heat-killed *C*. *albicans* (Fig 7I) and live *C*.*albicans* (Fig 7J), when compared with WT macrophages. Notably, administration with exogenous recombinant murine PGRN protein could significantly down-regulate the increased ability of PGRN KO macrophages to phagocytose live *C*. *albicans*. PGRN KO macrophages also displayed increased phagosome formation upon *C*.*albicans* infection by scanning electron microscopy and transmission electron microscopy analysis (Fig 7K). Furthermore, PGRN KO macrophages were significantly more efficient in the killing of live *C*. *albicans* than WT cells, and exogenous recombinant murine PGRN protein could significantly decrease the killing rate of PGRN KO macrophages (Fig 7L). We also found that PGRN loss potently enhanced the production of reactive oxygen species (ROS) by *C*. *albicans*-stimulated macrophages (Figs 7M and S4A). Besides, the increase of phagocytosis and killing rate was accompanied with significantly increased TNF-α and IL-15 production, but with significantly decreased IL-6 and IL-1β production in PGRN KO macrophages upon *C*. *albicans* infection when compared with WT cells (Fig 7N). In PGRN KO neutrophils, the phagocytic activity (Fig 7O and 7P), phagosome formation (Fig 7Q), fungal killing (Fig 7R), and ROS production (Figs 7S and S4B) were enhanced upon *C*. *albicans* infection compared with WT cells, while treatment with recombinant murine PGRN could reverse the increase of phagocytosis and killing rate in PGRN KO neutrophils (Fig 7P–7R). We also determined whether PGRN is involved in NETs release upon *C*. *albicans* infection, and found that NETs release was significantly increased in PGRN KO neutrophils compared with WT cells (Fig 7T and 7U). Besides, PGRN-deficient neutrophils produced significantly more TNF-α and less IL-6 than WT neutrophils upon *C*. *albicans* infection (Fig 7V).

### PGRN negatively regulated Dectin-2 expression and Syk activation in macrophages and neutrophils

**Fig 8:**
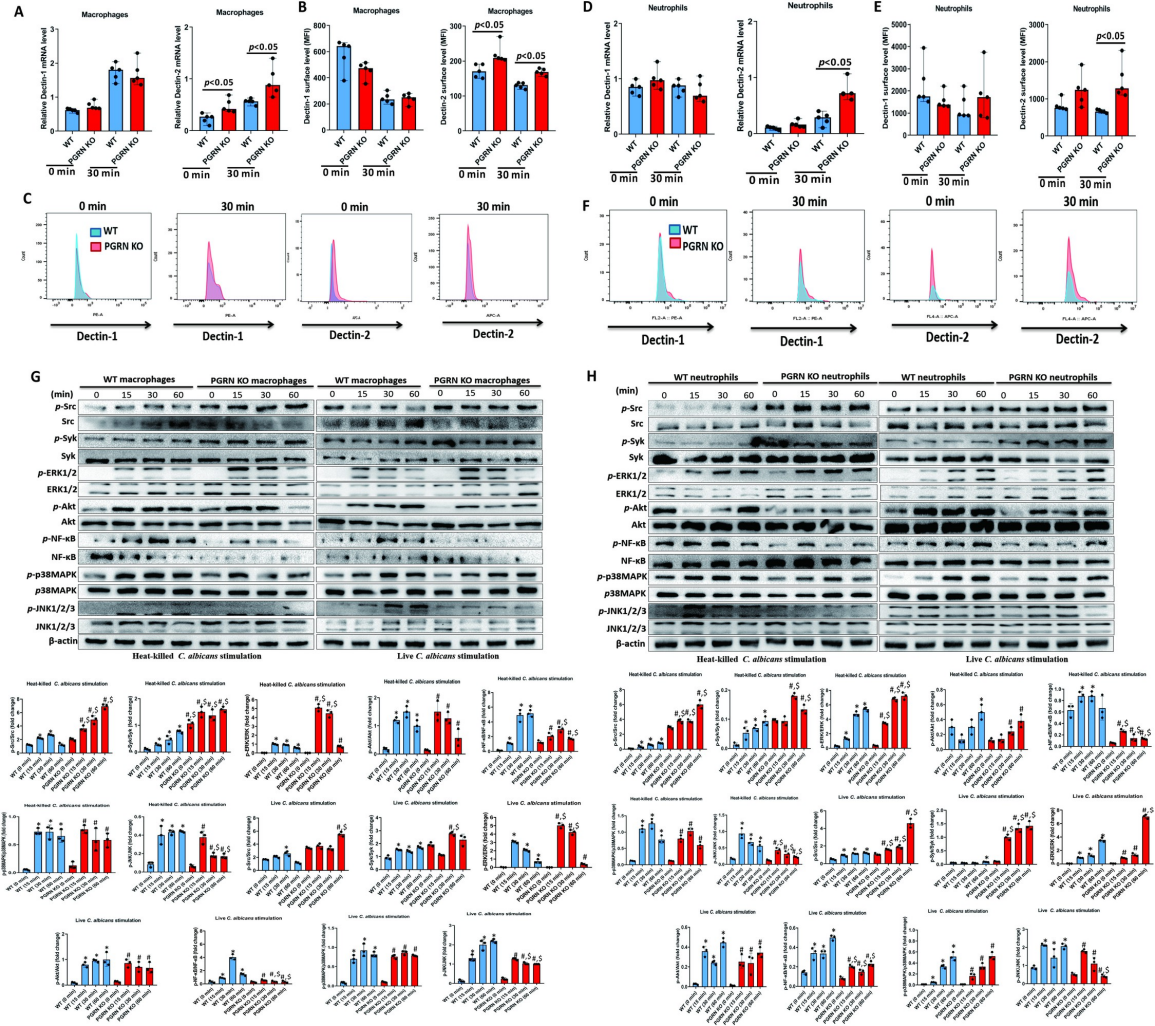
PGRN deletion enhanced Dectin-2 expression and Syk phosphorylation in macrophages and neutrophils. (A) mRNA expression levels of Dectin-1 and Dectin-2 in WT (n = 5) and PGRN-deficient (n = 5) BMDM with and without heat-killed *C*. *albicans* yeast stimulation (at a multiplicity of infection of 1:10). The Mann–Whitney *U* test was used to analyze the difference between groups denoted by horizontal lines. (B) Surface expression levels of Dectin-1 and Dectin-2 in WT (n = 5) and PGRN-deficient (n = 5) BMDM with and without heat-killed *C*. *albicans* yeast stimulation (at a multiplicity of infection of 1:10). The Mann–Whitney *U* test was used to analyze the difference between groups denoted by horizontal lines. MFI = mean fluorescence intensity. (C) Representative figures of Dectin-1 and Dectin-2 expression on BMDM (n = 5) analyzed by surface staining and flow cytometry. (D) mRNA expression levels of Dectin-1 and Dectin-2 in WT (n = 5) and PGRN-deficient (n = 5) neutrophils with or without heat-killed *C*. *albicans* yeast stimulation (at a multiplicity of infection of 1:10). The Mann–Whitney *U* test was used to analyze the difference between groups denoted by horizontal lines. (E) Surface expression levels of Dectin-1 and Dectin-2 in WT (n = 5) and PGRN-deficient (n = 5) neutrophils with or without heat-killed *C*. *albicans* yeast stimulation (at a multiplicity of infection of 1:10). The Mann–Whitney *U* test was used to analyze the difference between groups denoted by horizontal lines. (F) Representative figures of Dectin-1 and Dectin-2 expression on neutrophils (n = 5) analyzed by surface staining and flow cytometry. (G) WT or PGRN-deficient BMDM were stimulated with either live or heat-killed *C*. *albicans* (at a multiplicity of infection of 1:10) for the indicated times, and phosphorylation levels of Src, Syk, Akt, ERK1/2, NF-κB-p65, JNK and p38MAPK were determined with phosphospecific antibodies by Western blot analysis. The intensity of the proteins was measured, and data from three independent experiments were analyzed with a one- way ANOVA followed by Tukey’s test. *p<0.05 when compared with WT cells at 0 min. ^#^*p*<0.05 when compared with PGRN KO cells at 0 min. ^$^*p*<0.05 when compared between PGRN KO cells and WT cells at the same time point. (H) WT or PGRN-deficient neutrophils were stimulated with either live or heat-killed *C*. *albicans* (at a multiplicity of infection of 1:10) for the indicated times, and phosphorylation levels of Src, Syk, Akt, ERK1/2, NF-κB-p65, JNK and p38MAPK were determined with phosphospecific antibodies by Western blot analysis. The intensity of the proteins was measured, and data from three independent experiments were analyzed with a one-way ANOVA followed by Tukey’s test. *p<0.05 when compared with WT cells at 0 min. ^#^*p*<0.05 when compared with PGRN KO cells at 0 min. ^$^*p*<0.05 when compared between WT cells at the same time point.

We next investigated the mechanism(s) underlying the increased antifungal activity of PGRN-deficient macrophages and neutrophils, and found that mRNA expression levels of Dectin-2, but not Dectin-1, were significantly increased in PGRN-deficient macrophages when compared with WT cells (Fig 8A). Flow cytometry analysis confirmed that the protein expression levels of Dectin-2, but not Dectin-1, on the surface of PGRN KO macrophages were significantly enhanced (Fig 8B and 8C). Similarly, we also observed comparable expression of Dectin-1 in PGRN KO and WT neutrophils (Fig 8D–8F). However, the mRNA expression and surface protein levels of Dectin-2 were up-regulated in PGRN KO neutrophils, which reached statistical significance at 30 min after *C*. *albicans* infection compared to WT neutrophils (Fig 8D–8E). Furthermore, supplementation with recombinant murine PGRN protein could significantly down-regulate the surface expression of Dectin-2 on macrophages and neutrophils (S5 Fig).

We then analyzed the CLR signaling pathways involving the Src family member kinase activity, tyrosine kinase Syk, protein kinase B (Akt), extracellular signal-regulated kinase (ERK), nuclear factor kappa-B (NF-κB), c-Jun N-terminal kinase (JNK) and p38 mitogen-activated protein kinase (MAPK) signaling. The levels of Src kinase activation following the stimulation of macrophages and neutrophils with heat-killed *C*. *albicans* or live *C*. *albicans* were increased in PGRN KO cells compared with WT cells at various time points (Fig 8G and 8H). Src-dependent phosphorylation of Syk in PGRN-deficient macrophages and neutrophils was also enhanced at various time points (Fig 8G and 8H). Additionally, phosphorylation of the downstream molecular ERK was increased in PGRN-deficient macrophages and neutrophils at various time points. However, the activation of NF-κB and JNK was attenuated in PGRN KO cells compared with WT cells at various time points. Besides, the phosphorylation levels of Akt and p38MAPK were similar at all time points. These data indicated that PGRN negatively controlled ERK signaling downstream of Src and Syk in macrophages and neutrophils, in response to Dectin-2 engagement.

### PGRN loss enhanced defense response against fungal infection in macrophages and neutrophils on the transcriptome level

To further identify target gene(s) that may be responsible for antifungal activity of PGRN-deficient macrophages and neutrophils at the transcriptional level, we performed RNA sequencing analysis with purified PGRN KO and WT BMDM or neutrophils after *C*. *albicans* stimulation (Gene Expression Omnibus database; accession number GSE201167. To review GEO accession GSE201167: Go to https://www.ncbi.nlm.nih.gov/geo/query/acc.cgi?acc=GSE201167)). We found that defense response to other organism (including fungus) was the top 1 gene set that was up-regulated in PGRN KO macrophages (S6A–S6C Fig), and several significantly up-regulated genes have been reported to be associated with antifungal activity, such as IL-15RA (interleukin-15 receptor subunit alpha) [25], IFI44 (interferon associated protein) [26], CD69 [27], and CSF1 (macrophage colony-stimulating factor) [28] (S6D Fig). In PGRN KO neutrophils, genes involved in defense response to fungus were also up-regulated after *C*. *albicans* stimulation (S6E–S6G Fig), and we observed several significantly up-regulated genes that have been reported to be associated with antifungal activity (S6H Fig), such as Elane (elastase) [29], Mpo (myeloperoxidase) [30], C4b (Complement 4b) [31], Mzb1 (marginal zone B and B-1 cell-specific protein) [32], Pou2af1 (POU domain class 2-associating factor 1) [33], Tslp (thymic stromal lymphopoietin) [34], Epx (eosinophil peroxidase) [35], and Epcam (epithelial cell adhesion molecule) [36]. These transcriptome differences suggest PGRN loss up-regulated antifungal activity in both macrophages and neutrophils *in vitro*.

### PGRN impaired phagocytosis and killing of C. albicans in human macrophages and neutrophils

**Fig 9:**
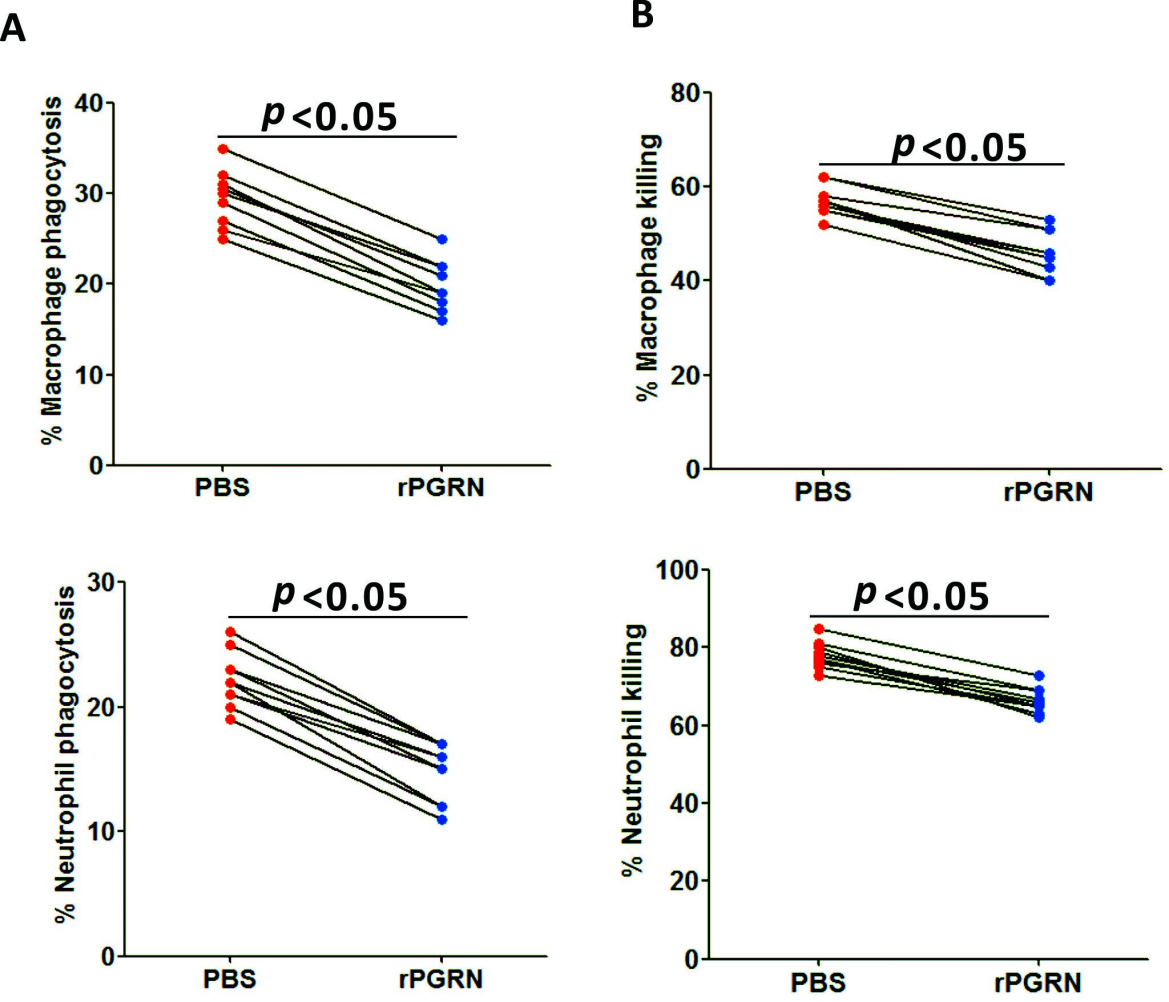
PGRN enhanced the phagocytic and killing activity of human macrophages and neutrophils in response to fungal infection. (A) Human monocyte-derived macrophages and neutrophils isolated from 10 healthy volunteers were preincubated with recombinant human PGRN (100 ng/ml) for 2 hours and challenged with FITC-labeled heat-killed *C*. *albicans* yeast at a multiplicity infection of 1:2 for 45 minutes, and the percentage of fungal phagocytosis was calculated. (B) Human monocyte-derived macrophages and neutrophils isolated from 10 healthy volunteers were preincubated with recombinant human PGRN (100 ng/ml) for 2 hours and challenged with live *C*. *albicans* at a multiplicity infection of 1:10 overnight, and the percentage of fungal killing was calculated. Wilcoxon signed-rank test was used to analyze the statistical difference, and *p* values were shown when compared between groups denoted by horizontal lines.

To translate murine findings into human beings, we investigated whether treatment with recombinant human PGRN protein could affect antifungal activity of human macrophages and neutrophils. We observed that recombinant human PGRN protein could significantly decrease the ability of human macrophages and neutrophils to phagocytize FITC-labeled *C*. *albicans* and to kill live *C*. *albicans* (Fig 9A and 9B).

## Discussion

**Fig 10:**
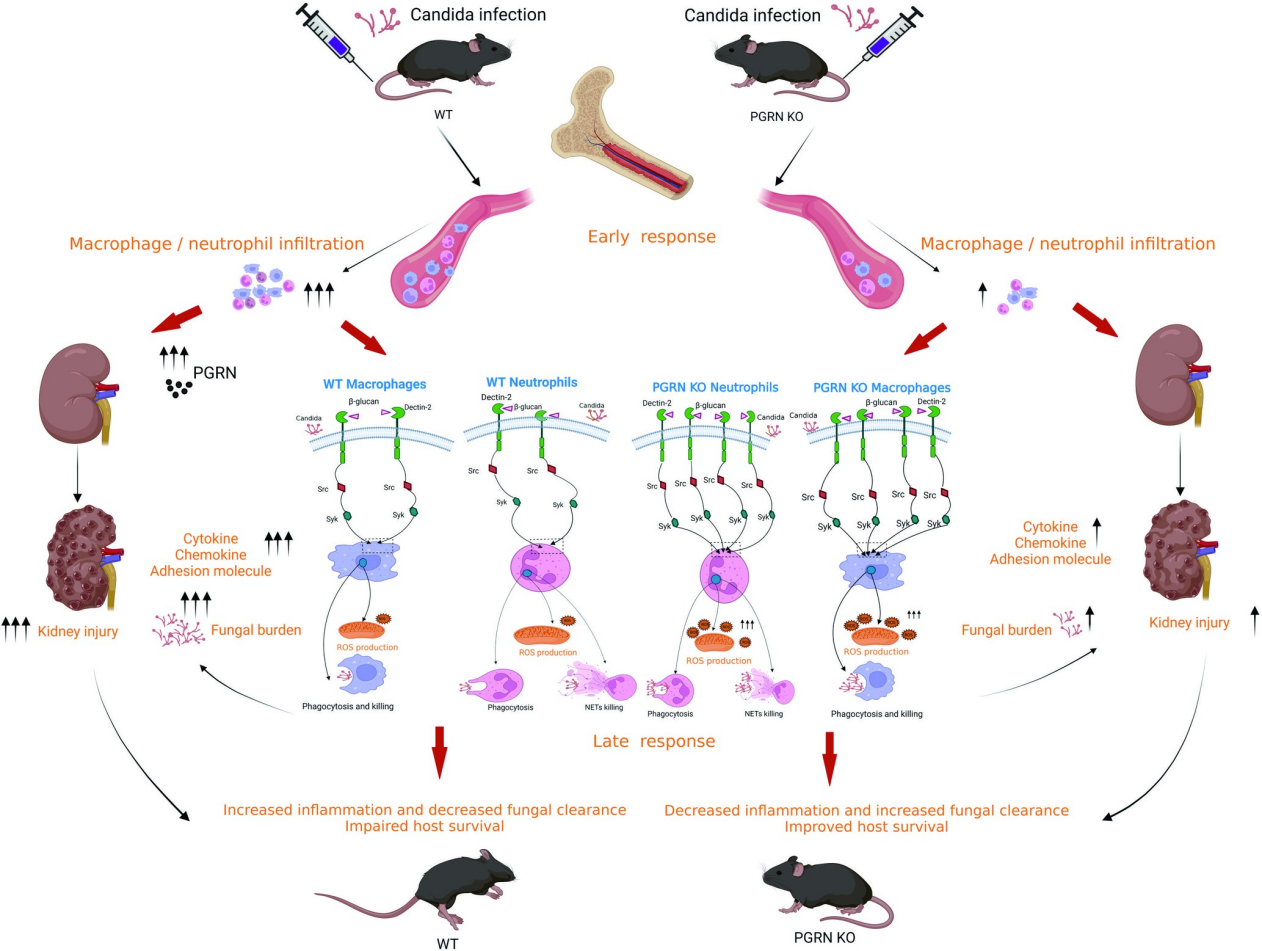
Proposed model of PGRN regulation of inflammatory reactions and antifungal immunity in response to *C*. *albicans* infection. *C*. *albicans* recognition induces PGRN production, which plays an important role in the immunopathology of invasive *C*. *albicans* infection. At an early phase of invasive *C*. *albicans* infection, PGRN loss does not affect fungal burden in the kidney, but it dampens acute inflammatory responses in the kidney involving the production of lower levels of inflammatory cytokine (IL-6) and chemokine (CXCL1 and CCL2), and the expression of reduced levels of adhesion molecule (ICAM-1 and P-Selectin), as well as the infiltrate of declined numbers of macrophage and neutrophil, supporting that PGRN participates in modulating an early harmful inflammatory response to *C*. *albicans* infection. At a later phase of invasive *C*. *albicans* infection, PGRN loss leads to lower fungal burden in the kidney, which is associated with increased antifungal immunity in PGRN-deficient macrophages and neutrophils. In the absence of PGRN, it can induce up-regulated expression of Dectin-2, and enhance phosphorylation of Src and Syk, as well as increase phagocytosis, reactive oxygen species (ROS) production, neutrophil extracellular traps (NETs) release, and fungal killing in macrophages or neutrophils, which limits fungal load and provides less inflammatory stimulus. Therefore, PGRN loss contributes to the protection against lethal *C*. *albicans* sepsis at two different but interconnected ways: the attenuated harmful host inflammatory response and the enhanced antifungal immunity that eliminates the *C*. *albicans*. These two processes regulate each other, which improves host survival in PGRN-deficient mice.

*C*. *albicans* sepsis is associated with one of the highest rates of mortality, and novel antifungal drugs for death reduction are urgently required. By harnessing the experimental model of invasive *C*. *albicans* infection in mice, we here showed that PGRN release was increased in mice with systemic candidiasis. PGRN loss could protect mice from a lethal systemic infection with *C*. *albicans*, and PGRN deficiency in hematopoietic cell compartment was important for this process. PGRN neutralization by anti-PGRN antibody at the onset of *C*. *albicans* sepsis protected mice from morbidity and mortality. Interestingly, at an early phase (days 1, 4, and 7) of invasive *C*. *albicans* infection, PGRN KO mice exhibited similar fungal burdens in the kidneys, as well as spleens, lungs and brains, when compared with WT animals, whereas, PGRN deficiency conferred decreased inflammatory responses in the kidneys that involved the production of lower levels of inflammatory cytokine (IL-6) and chemokine (CXCL1 and CCL2), the expression of reduced levels of adhesion molecule (ICAM-1 and P-selectin), and the infiltrate of declined numbers of macrophage and neutrophil, indicating that PGRN deficiency decreased *Candida*-induced early and acute inflammation and immunopathology. At a later phase of infection (day 9), fungal burden in the kidneys was significantly lower in PGRN-deficient mice than in control WT mice, suggesting that PGRN deletion also facilitated fungal clearance in systemic candidiasis, which was associated with increased antifungal immunity of PGRN-deficient macrophages and neutrophils. Furthermore, PGRN loss led to enhanced surface expression of Dectin-2 on macrophages and neutrophils, which was accompanied by an increase of Src and Syk phosphorylation and an enhancement of downstream ERK signaling upon *C*. *albicans* stimulation. Therefore, these results suggest that PGRN deletion protected host from IC by simultaneously reducing renal inflammation and limiting fungal load, which could ameliorate inflammation-induced renal damage and improve host survival. Our collective observations are illustrated in Fig 10 as a working hypothesis.

For the first time, to the best of our knowledge, we have revealed that PGRN acted as a detrimental factor of host immune response to IC. The PGRN-deficient mice infected with *C*. *albicans* exhibited decreased mortality that was associated with a dampened early and acute inflammatory response. A variety of studies have shown that exacerbated inflammatory immune reactions were an important cause of tissue damage and death in the murine *Candida* infection model [19–22]. Despite that PGRN KO mice had similar fungal burden in the kidney, which is the primary infected organ in the systemic candidiasis mouse model, at the early times of *C*. *albicans* infection (days 1, 4, and 7), the production of proinflammatory cytokine IL-6, a major cause of sepsis [37,38], in PGRN-deficient mice was significantly lower than that in WT animals. PGRN deletion also decreased the production of the macrophage and neutrophil chemokines CCL2 and CXCL1, and reduced the expression of adhesion molecules ICAM-1 and P-Selectin in the kidneys at early phases of infection, paralleling the decrease of macrophage and neutrophil recruitment to the infected kidneys in PGRN-deficient mice after IC. These findings are therefore in agreement with our previous data observed in the lungs of PGRN-deficient mice after bacterial or influenza virus infection [14,16]. It has been shown that pharmacological suppression of monocytes/macophages and neutrophils could protect against *C*. *albicans* infection [39]. Thus, the early decrease in inflammatory mediator production and macrophage/neutrophil recruitment observed in PGRN-deficient mice might contribute to the improvement in renal function and survival after *C*. *albicans* infection. On the other hand, PGRN-deficient mice displayed significantly higher production of INF-γ, IL-17A and IL-23 at early times after *C*. *albicans* infection, and these cytokines have been identified as protective factors against IC [21,23,40,41]. In fact, PGRN has been shown to be able to inhibit Th1 and Th17 cells, and significantly lower levels of INF-γ and IL-17 were observed in PGRN-deficient mice compared with WT mice in experimental autoimmune encephalomyelitis, experimental autoimmune uveitis or coxsackievirus-B3-induced viral myocarditis [42–44]. The kidney fungal load has also been shown to correlate with the severity of renal injury and the progress of *C*. *albicans* sepsis [6,7,19]. At a later phase of *C*. *albicans* infection (day 9), we did find that PGRN-deficient mice had significantly lower fungal loads in the kidneys. The decreased fungal loads, which provided less inflammatory stimulus, may be able to further reduce renal inflammation and injury after *C*. *albicans* infection. In addition to IL-6, CCL2, and CXCL1, we found that the release of proinflammatory cytokine IL-1β was significantly reduced in the kidneys of PGRN KO mice when compared with WT animals at day 9 after *C*. *albicans* infection. Collectively, we can conclude that PGRN loss contributed to protection against lethal *C*. *albicans* sepsis at two different but interconnected ways: the attenuated harmful host inflammatory response and the enhanced host antifungal immunity that eliminates the *C*. *albicans*. These two processes regulated each other, which were responsible for the decreased mortality observed in PGRN-deficient mice after IC.

Another important finding of the present study is that PGRN deficiency enhanced phagocytosis, phagosome formation, ROS production and fungal killing in macrophages and neutrophils, and PGRN loss also increased the formation of NETs in neutrophils. Recognition of β-glucans and α-mannans on invading fungi via CLRs Dectin-1 and Dectin-2, respectively, is crucial for phagocytosis and killing in macrophages and neutrophils [45,46]. Despite its name, Dectin-2 is only 20–25% homologous to Dectin-1 [47]. Here we found that PGRN loss resulted in up-regulated expression of Dectin-2, but not Dectin-1, in both macrophages and neutrophils. A previous study has demonstrated that WT and Dectin-1 KO mice were equally susceptible to candida infection [48], while Dectin-2 KO mice were more susceptible to systemic candidiasis [49]. Unlike Dectin-1, Dectin-2 activates Syk signaling, through an immunoreceptor tyrosine-based activation motif (ITAM)–bearing Fc receptor γ (FcRγ) signaling chain [50]. We found that Src activation and Syk phosphorylation were enhanced in PGRN-deficient macrophages and neutrophils compared with WT cells upon *C*. *albicans* infection. Syk signaling has been shown to play an essential role in macrophage and neutrophil fungicidal activity against Candida species, such as ROS generation, phagosome formation, phagocytosis, killing, or NETs release [10,11]. Besides, phosphorylation of the downstream molecular ERK was enhanced in PGRN-deficient macrophages and neutrophils upon *C*. *albicans* infection, which may account for increased phagocytosis and killing ability, as well as enhanced TNF-α and IL-15 secretion. ERK activation has been shown to be critical for the survival of *C*. *albicans*-infected mice [51]. However, we found that the activation of NF-κB and JNK signaling was down-regulated, which may be implicated in the lower production of IL-6 or IL-1β from PGRN KO macrophages and neutrophils compared to WT cells upon *C*. *albicans* infection. A previous study has shown that JNK1 negatively controlled antifungal immunity during *C*. *albicans* sepsis [7]. Therefore, PGRN differentially regulated the activation of intracellular signaling pathways, which may participate in antifungal immunity and inflammatory response of macrophages and neutrophils. Anyway, our observations suggest that PGRN loss facilitated fungus elimination by macrophages and neutrophils, which is likely due to enhanced activation of Dectin-2-Syk signaling cascade. RNA sequencing further demonstrated that genes involved in antifungal immune response were highly up-regulated in PGRN-deficient macrophages and neutrophils upon *C*. *albicans* infection, indicating that the lack of PGRN facilitated fungus elimination by enhancing antifungal immunity of macrophages and neutrophils, which would constrain fungal growth in the kidney, thereby limiting the excessive inflammation and improving mouse survival after IC.

TLRs as a family of pattern recognition receptors (PRRs) of the immune system play a crucial role in the recognition of fungal pathogens such as *C*.*albicans* [52,53], and TLRs activation induces the synthesis of proinflammatory molecules. Here we found that TLR4 was important for the production of PGRN after *C*.*albicans* infection, which is consistent with previous data showing that TLR4 mediated proinflammatory cytokine (TNF-α and IFN-γ) induction after *Candida* stimulation [54]. Thus, future studies should aim to investigate TLR4-related mechanisms regulating PGRN expression in response to *C*.*albicans*. Another interesting new question arises from our data that Dectin-2 expression was enhanced in PGRN-deficient macrophages and neutrophils upon *C*.*albicans* infection. Considering the crucial role of dectin-2 in host defense against *C*. *albicans* [45,46], the factors mediating Dectin-2 expression by PGRN should be further investigated, especially using Dectin-2 KO mice. Explanation of such questions may reveal other novel mediators of immunopathology in this model and could provide further insight into balance between the response of inflammation and the control of fungal burden in IC.

In conclusion, our study uncovers PGRN as a central mediator in the immunopathology of IC, as it promotes early inflammatory response and impairs antifungal immunity in macrophages and neutrophils. Therefore, targeting PGRN could serve as a novel treatment for fungal infection, which might act as an alternative approach to current antifungal drugs for further amelioration of disease outcome in the patients with IC.

## Methods

### Ethics statement

All animal experiments were discussed with and approved by the Animal Care and Use Committee of the Chongqing Medical University and carried out according to the recommendations in the guide for the care and use of laboratory animals conformed to animal protection laws of China and applicable guidelines (YSDWBHF-R-27/08/2009).

### Mice

PGRN KO mice, TLR2 KO mice, TLR4 KO mice, TLR7 KO mice, and IFNAR KO mice raised on the C57BL/6 background were purchased from the Jackson Laboratory (Bar Harbor, ME). All mice were genotyped by PCR using genomic tail DNA. WT C57BL/6 mice were purchased from Chongqing Medical University. All mice were housed in the specific pathogen-free animal facility at Chongqing Medical University. In all experiments described here, sex- and age-matched mice were used.

### Bone marrow (BM)-chimeric mice

Six-week-old recipient mice were lethally irradiated by X-ray (550 rad × 2) and administered with 8 x 10^6^ bone marrow cells via tail vein injection within 1 hour. Chimaeras were then used for subsequent experiments at 8 weeks after the initial reconstitution.

### C. albicans sepsis model

Mice (6–8 weeks of age) were injected via lateral tail veins with 200 μl of a suspension containing different doses of *C*. *albicans* (SC5314) in sterile PBS (Hyclone). The *C*. *albicans* cells were grown overnight at 30°C in extract-peptone-dextrose (YPD) medium for yeast formation. Cells were harvested, washed twice in phosphate-buffered saline (PBS) and counted before intravenous infection. After *C*. *albicans* infection, the survival rates were monitored for 40 days. For fungal burden determinations, the tissues were weighed, homogenized in PBS and plated on the YPD plates in a series of diluted solutions. After 48 hours of incubation at 37°C, the colony-forming units (CFU) were counted and determined as CFU/g tissue.

### Determination of M-CASS score

M-CASS scores were determined according to eight markers: fur aspect, activity, posture, behavior, chest movements, chest sounds, eyelids, and weight loss. A definition of each parameter was completed as described previously [55], and each mouse were scored from 1 to 4.

### Histopathology and immunohistochemistry

Mice were euthanized post-infection, and the kidneys were removed, fixed with 10% formalin, dehydrated in ethanol and embedded in paraffin. The tissue sections (3–5μM) were mounted on glass slides, processed for hematoxylin and eosin (H&E) and Calcofluro White (CW) staining. Two independent pathologists scored the histology in a blinded manner, and the pathology scores of kidneys were determined according to the following variables: number of thrombi, number of (micro) abscesses, presence and degree of inflammation, and presence and degree of necrosis [7,13]. The fungal burden in the kidney was based upon CW staining as described previously [56]. To detect apoptotic cells, TUNEL assay was done on frozen kidney sections using the TUNEL apoptosis detection kit according to manufacturer’s protocol (Millipore, Temecula CA). The number of TUNEL-positive cells was counted in 15 randomly selected high powered fields (400X) per slide.

### Measurement of serum creatinine and blood urea

Serum was collected by retro-orbital bleeding at the indicated times after *C*. *albicans* infection. Blood creatinine and urea nitrogen levels were measured according to the protocols of the International Federation of Clinical Chemistry, by spectro-photometric analysis (modular DDP, Roche).

### Measurement of cytokines and chemokines

The protein levels of progranulin (PGRN) were determined by mouse PGRN enzyme linked immunosorbent assay (ELISA) kit (RayBiotech). Assessment of inflammatory cytokines or chemokines including TNF-α, IL-6, IFN-γ, GM-CSF, IL-1β, IL-2, IL-10, IL-12, IL-15, IL-17A, IL-18, IL-23, CXCL1 and CCL2 was performed by using ELISA kits (Biolegend) according to the manufacturers’ instructions.

### Flow cytometry analysis

Cell suspensions were surface labeled with fluoro-chrome-conjugated antibodies for 10 minutes at 4°C in staining buffer (PBS containing 1% BSA). The following antibodies were used for flow cytometry analysis: anti-CD11b Alexa Fluor 647 (clone M1/70, BD pharmingen), anti-F4/80 phycoerythrin (PE) (clone T45-2342, BD pharmingen), anti-Ly-6G fluoresceine isothiocyanate (FITC) (clone RB6-8C5, BD pharmingen), anti–Dectin-1 PE (clone RH1, Biolegend), and anti–Dectin-2 allophycocyanin (APC) (catalog FAB1525A, R&D Systems). Data were acquired using a FACSAria (BD Biosciences) and analysed using the FlowJo software (Tree Star). The absolute numbers of CD11b^+^Ly-6G^+^ neutrophils and CD11b^+^F4/80^+^ macrophages were then quantified by dividing the number of positive cellular events by the number of bead events and multiplying the result by the bead concentration using a FACScan flow cytometer (BD Biosciences). Absolute numbers were derived from the pooled sample of 5 biopsy specimens. Expression of cell surface Dectin-1 and Dectin-2 receptors on 5,000 viable cells was quantitatively analyzed by flow cytometry (BD Biosciences) in terms of mean fluorescence intensity (MFI).

### In vivo administration of anti-PGRN antibodies

PGRN neutralization was performed by intraperitoneally (i.p.) administration of 5 μg of anti-PGRN monoclonal antibodies (Clone 333731, R&D systems) on day 0 (same day as *C*. *albicans* infection), followed by a booster dose of 2.5 μg at 24 hours later after invasive *C*. *albicans* infection. Rat IgG was delivered in a similar way as control.

### Macrophage and neutrophil depletion assay

For macrophage depletion, the clodronate-encapsulated liposomes and PBS-encapsulated liposomes were prepared as described previously [57,58]. Clodronate-encapsulated liposomes were delivered i.p. (200 μL) to deplete macrophages. PBS-encapsulated liposomes were delivered in a similar fashion as a control.

For neutrophil depletion, each mouse was injected i.p. with 100 μg of anti-mouse Ly-6G antibodies (Clone 1A8, Biolegend) before *C*. *albicans* infection, whereas control mice were treated with nonspecific rat IgG (Biolegend). The macrophage and neutrophil depletion efficacy was performed at 24 hours after treatment by flow cytometry on the basis of expression of F4/80 and Ly-6G, respectively.

### Preparation of bone marrow-derived macrophages (BMDM) and neutrophils

For BMDM preparation, bone marrow was collected from femurs and tibias by flushing with Roswell Park Memorial Institute (RPMI) 1640 Media. Cells were washed and resuspended in Dulbecco’s Modified Eagle Medium (DMEM) supplemented with 10% FBS, 100 U/ml penicillin, and 100 mg/ml streptomycin plus 10% conditioned medium of L929 cells (a source of M-CSF) at 37°C for 5 days. After culture, 90% of cells were F4/80^+^ macrophages.

For neutrophil preparation, mouse bone marrow and splenic neutrophils were isolated from tibias and femurs of mice using anti–Ly-6G magnetic beads (Miltenyi Biotec). After centrifugation, 95% of cells were Ly-6G^+^ neutrophils.

### Phagocytosis assay

The phagocytosis assay of heat-killed *C*. *albicans* (HKCAs) was performed as described previously with minor modification [6,24]. Briefly, HKCAs were labeled with FITC (Sigma-Aldrich) in 100 mM N-2-hydroxyethylpiperazine-N-ethane-sulphonicacid (HEPES) buffer (pH 7.5) for 10 minutes. Subsequently, BMDM or neutrophils were cocultured with FITC-labeled HKCAs (at a multiplicity of infection of 1:2) at 37°C for 45 minutes. Adherent fungal cells were quenched with trypan blue (Sigma Aldrich), and phagocytosis was expressed as the percentage of phagocytosing FITC-labeled *C*. *albicans* or as the MFI.

For phagocytosis assay of live *C*. *albicans*, we used a procedure similar to that described previously [19,59]. Briefly, BMDM or neutrophils were seeded in 12-well plates, and live *C*. *albicans* was then added at a multiplicity of infection of 1:2 and incubated for 30 min at 37°C (in a 5% CO2 environment) to allow phagocytosis. Cells were then collected by cytospin (700 g for 7 min) and stained by Hemacolor. The fungal cell phagocytosis was expressed according to the following formula: percentage of phagocytosis = number of cells containing one or more fungal cells/100 cells counted. In some experiments, cells were pretreated with or without recombinant murine PGRN protein for 1 hour (catalog 2557-PG-050, R&D systems).

### Killing assay

To assess the fungicidal activity, BMDM and neutrophils were plated in replicates at a density of 1 x 10^5^ cells/well in 96-well plates, and then cells were incubated with live *C*. *albicans* at a multiplicity of infection of 1:10 incubated overnight at 37°C. After incubation, BMDM and neutrophils were fixed by the addition of paraformaldehyde at a final concentration of 2%. Subsequently, *C*. *albicans* were stained with Crystal Violet (Sigma) and killing was assessed by a comparison of colony numbers in wells with or without BMDM or neutrophils. In some experiments, cells were pretreated with or without recombinant murine PGRN protein for 2 hours.

### Electron microscopy

BMDM and neutrophils were challenged with heat-killed *C*. *albicans* (at a multiplicity of infection of 1:20) for 30 minutes at 37°C. For transmission electron microscopy (TEM), macrophages and neutrophils were plated on bacteriologic plastic and fixed with the use of a mixture of 2.5% glutaraldehyde, 2% paraformaldehyde, and 0.1% picric acid in 100mM cacodylate buffer (pH 7.0) containing 2mM EGTA and 1mM MgCl2. The samples were post-fixed in 1% osmium in 100mM cacodylate buffer (pH 7.0) for 1 hour at 4°C, washed with distilled water, and stained en bloc with 2% aqueous uranyl acetate for 2 hours at 4°C, in the dark. The samples were dehydrated with ethanol, and the cells were released from plastic using propylene oxide. The cells were pelleted and washed several times with propylene oxide and embedded in resin. Ultrathin (~70-nm thick) sections were cut, stained with uranyl acetate and lead citrate, and examined in a FEI Tecnai 12 electron microscope.

For scanning electron microscopy, cells were plated on coverslips and fixed as described in the paragraph above. Samples were rinsed several times with distilled water, dehydrated through a series of ethanol washes, and critical point-dried. After sputter-coating with gold, samples were examined in a JOEL JSM 5510 scanning electron microscope. Images were viewed with an FEI Tecnai 12 microscope (FEI UK Ltd) operating at 80 kV with a 20-μm objective aperture using a Gatan US1000 1 2k-2k CCD camera and Gatan Digital Micrograph software Version 3.11.1. Images were then analyzed.

### Detection of ROS production

BMDM and neutrophils were incubated with HKCAs (at a multiplicity of infection of 1:20) for 0, 2, 4 or 6 hours, to measure the total intracellular ROS levels, cells were treated with the fluorogenic probe H2DFFDA (Life Technologies) at 5 μM for 30 min at 37°C. The medium was then removed, and the cells were returned to prewarmed fresh growth medium. The emitted fluorescence was detected by a fluorescent microplate reader using 490/520 nm excitation/emission filters (Molecular Devices, Sunnyvale, CA). The ROS levels are reported as fluorescence intensity.

### NETs formation quantification

This procedure was performed as previously described [60]. Briefly, neutrophils (5 x 10^5^ cells) were stimulated with live *C*. *albicans* (at a multiplicity of infection of 1:10) at 37°C. An antibody bound to a 96-well clear-bottomed black plate captured the enzyme myeloperoxidase (MPO, 5 μg/mL; Abcam). According to the manufacturer’s instructions, the amount of DNA bound to the enzyme was quantified using the Quant-iT PicoGreen Kit (Invitrogen). The fluorescence intensity (excitation at 488 nm and emission at 525 nm) was quantified using a FlexStation 3 Micro-plate Reader (Molecular Devices).

### Quantitative reverse transcription polymerase chain reaction (PCR)

Total RNA was isolated using 1 ml of TRIzol (Takara) according to the manufacturer’s protocol. RNA (500 ng) was reverse transcribed with PrimeScrip RT Master Mix. Quantitative PCR was performed using SYBR Premix Ex Taq II and the following primers: murine dectin-1 (Forward): 5’-GACCCAAGCTACTTCCTC-3’, murine dectin-1 (Reverse): 5’-GCAGCACCTTTGTCATACT-3’; murine dectin-2 (Forward): 5’-ACCCCTGACCTTCTGAACATACAC-3’, murine dectin-2 (Reverse): 5’-TGAGCCCCCATCTGAACACA-3’; murine KIM-1 (Forward): 5’-CTGCTGCTACTGCTCCTTGT-3’, murine KIM-1 (Reverse): 5’-GCAACCACGCTTAGAGATGC-3’; murine ICAM-1 (Forward): 5’-TGGATACCTGAGCATCACCA-3’, murine ICAM-1 (Reverse): 5’-CTGCTACCTGCACTTTGCC-3’; murine P-Selectin (Forward): 5’-GAACAATCCAGGTTGCCTTG-3’, murine P-Selectin (Reverse): 5’-CAGTTCATGTGCGATGAAGG-3’; murine GAPDH (forward): 5’**-**AGGTCGGTGTGAACGGATTTG-3’, murine GAPDH (reverse): 5’**-**TGTAGACCATGTAGTTGAGGTCA-3’ Relative quantification was performed with the ΔΔCt-method. Expression level of the genes of interest was normalised to the expression level of the housekeeping gene to GAPDH, which was utilized as a reference gene. Each data point was examined for integrity by analysis of the amplification plot.

### Western blot analysis

Cells were washed with ice-cold PBS and lysed in 0.2 ml lysis buffer (20 mM Tris–HCl, pH 8.0, 120 mM NaCl, 1% Triton X-100, 10 mM EDTA, 1 mM EGTA, 0.05% 2-mercaptoethanol, 1 x protease inhibitors). Cell debris was removed by centrifugation at 14 000 g for 15 min, and the supernatant was boiled in Laemmli sample buffer (Bio-Rad Laboratory, Hercules, CA) for 5 min. An equal amount of protein (10 μg) was subjected to sodium dodecyl sulfate (SDS)-polyacrylamide gel electrophoresis (PAGE) before blotting onto a polyvinylidene fluoride (PVDF) membrane (Amersham and Pharmacia Biotech). The membrane was blocked with 5% skimmed milk in Trisbuffered saline with 0.05% Tween-20 for 1 hour at room temperature, and probed with phospho-Syk^Tyr352^ antibody (catalog 2717; Cell Signaling Technology), Syk antibody (catalog 80460; Cell Signaling Technology), phospho-Src^Tyr527^ antibody (catalog 2105; Cell Signaling Technology), Src antibody (catalog 2109; Cell Signaling Technology), phospho-Akt^Ser473^ antibody (catalog 4060; Cell Signaling Technology), Akt antibody (catalog 9272; Cell Signaling Technology), phospho-ERK1/2^Thr202/Tyr204^ antibody (catalog 4370; Cell Signaling Technology), ERK1/2 antibody (catalog 4348; Cell Signaling Technology), phospho-JNK^Thr183/Tyr185^ antibody (catalog 4668; Cell Signaling Technology), JNK antibody (catalog 9252; Cell Signaling Technology), phospho-p38MAPK^Thr180/Tyr182^ antibody (catalog 4511; Cell Signaling Technology), p38MAPK antibody (catalog 8690; Cell Signaling Technology), phospho-NF-κB p65^Ser536^ antibody (catalog 3033; Cell Signaling Technology), NF-κB p65 antibody (catalog 8242; Cell Signaling Technology), or β-actin (catalog AF7017, Affinity) at 4° overnight. After washing, the membrane was incubated with corresponding secondary sheep anti-rabbit or sheep anti-mouse antibodies coupled to horseradish peroxidase (Amersham Pharmacia Biotech) for 1 hr at room temperature. Antibody–antigen complexes were then detected using an ECL chemiluminescent detection system. Protein bands were quantitated using Image J software (NIH, Bethesda, MD, USA), and band intensities were normalized to β-actin.

### RNA-sequencing and functional annotations

Total RNA was extracted from BMDM and neutrophils after *C*. *albicans* infection using QIAzol reagent (Qiagen Inc.) and the RNA samples were sent to Novogene Ltd., Beijing, China, for library preparation and transcriptomic sequencing with next-generation sequencing. mRNA was purified from total RNA (100 ng) using poly-T oligo-attached magnetic beads and fragmented randomly by the addition of fragmentation buffer for library construction. Sequencing libraries were created using NEBNext Ultra RNA Library Prep Kit for Illumina (Illumina Corp, San Diego, CA, USA). Libraries were sequenced on the Illumina NovaSeq 6000 platform to generate 150 bp paired-end reads according to the manufacturer’s instructions. The sequenced reads/raw reads containing low quality reads or reads with adaptor were filtered to obtain clean reads, and these clean reads were subsequently mapped to reference genome by HISAT (version 2.0.4). To analyze gene expression level, fragments per kilobase of transcript per million mapped reads were calculated by HTSeq (version 0.6.1). Differential gene expression analysis of eight experiments was performed using the DESeq2 (version 1.10.1, Bioconductor), genes with *P* < 0.05 regarded as differentially expressed. Differentially expressed genes were clustered by hierarchical clustering analysis and further annotated with GO enrichment analysis using ClustrProfiler (v3.8.1).

### Isolation of human monocytes and culture of monocyte-derived macrophages (MDM)

Human monocytes were isolated from peripheral blood of healthy volunteers as described previously [58,61], and they were collected into Becton Dickinson (Franklin Lakes, NJ) Vacutainer acid citrate dextrose tubes and differentiated into monocyte-derived macrophages (MDM) in 50 ng/ml macrophage colony-stimulating factor (M-CSF; Invitrogen) for 6 days. Cells were then washed in PBS and resuspended in prewarmed culture medium and used immediately for phagocytosis or killing assays in the presence or absence of recombinant human PGRN protein (catalog 2420-PG-050, R&D systems).

### Isolation of human peripheral blood neutrophils

Human peripheral blood neutrophils were isolated from fresh human buffy coat obtained from healthy volunteers. Whole blood was diluted with HBSS without divalent cations and layered over a discontinuous gradient cushion (Histopaque-1083; Sigma). The sample was spun for 30 min at 500g at room temperature. After discarding the supernatant fluid, the neutrophil/erythrocyte pellet was suspended in an equal volume of HBSS. The cell suspension was then diluted with dextran ([1.5%] final, m.w. 200,000–500,000 g/mol; Pharmacia, Uppsala, Sweden). The neutrophil-rich supernatant fluid was harvested and spun at 200g for 10 min. Contaminating erythrocytes in the pellet were removed by two sequential hypotonic lyses using 0.25 x PBS for 30 s followed by an equal volume of 1.75x PBS to restore isotonicity. The neutrophils were then counted. The final preparation of neutrophils was >98% pure as evaluated by morphological analysis. Trypan blue exclusion showed >96% viability in neutrophils. The isolated neutrophils were cultured in RPMI 1640 medium (Gibco Laboratories, Grand Island, NY) supplemented with 10% fetal bovine serum (Gibco) and 20 mM Hepes (Gibco). Cells were then washed in PBS and resuspended in prewarmed culture medium and used immediately for phagocytosis or killing assays in the presence or absence of recombinant human PGRN protein.

### Statistical analysis

Mice and cell data were expressed as mean ± standard deviation (SD). The Mann–Whitney *U* test or Kruskal-Wallis test followed by Dunn’s multiple comparisons post test was performed for data of non-normal distribution. Student’s *t* test or one-way analysis of variance (ANOVA) followed by Bonferroni post hoc analysis was used for data of normal distribution. For survival studies, Kaplan–Meier analyses followed by log-rank tests were performed. Data were plotted using Prism 8.0 (GraphPad Software). Statistical significance was set at *P* < 0.05.

## Supporting information

S1 Fig PGRN deficiency protected mice against invasive *C*.*albicans* infection. PGRN knockout (KO) and wild type (WT) mice were infected intravenously with 4 × 10^5^ colony forming units (CFU) of *C*. *albicans*. (A) Representative clinical appearance of WT and PGRN KO mice (n = 5 per group) after invasive *C*.*albicans* infection. (B) Anatomic pathology of kidneys in WT and PGRN KO mice (n = 5 per group) after invasive *C*.*albicans* infection. (C) Representative haematoxylin and eosin (HE) staining of kidney sections from WT and PGRN KO (n = 5 per group) mice after invasive *C*.*albicans* infection. Bottom panels are high magnification. Scale bars are 100 μM.
(TIF)

S2 Fig Effects of PGRN deficiency on cytokine and chemokine production upon invasive *C*.*albicans* infection. PGRN knockout (KO) and wild type (WT) mice were infected intravenously with 4 × 10^5^ colony forming units (CFU) of *C*. *albicans*. (A) Cytokines and chemokines in the kidneys (n = 5) were quantified by ELISA at the indicated times after *C*. *albicans* infection. (B) Cytokines and chemokines in the sera (n = 5) were quantified by ELISA at the indicated times after *C*. *albicans* infection. (C) Cytokines and chemokines in the livers (n = 5) were quantified by ELISA at the indicated times after *C*. *albicans* infection. (D) Cytokines and chemokines in the lungs (n = 5) were quantified by ELISA at the indicated times after *C*. *albicans* infection. (E) Cytokines and chemokines in the brains (n = 5) were quantified by ELISA at the indicated times after *C*. *albicans* infection. (F) Cytokines and chemokines in the spleens were quantified by ELISA at the indicated times after *C*. *albicans* infection.
(TIF)

S3 Fig PGRN deletion reduced infiltration of inflammatory macrophages and neutrophils in mice after invasive *C*.*albicans* infection. Flow cytometry gating scheme determining the percentage of mouse macrophages and neutrophils in the kidneys from PGRN KO and WT mice after *C*. *albicans* infection. Representative FACS plots and percentages of macrophages (CD11b^+^ F4/80^+^) and neutrophils (CD11b^+^ Ly-6G^+^) from five independent experiments were shown.
(TIF)

S4 Fig PGRN deficiency increased reactive oxygen species (ROS) production in macrophages and neutrophils. (A) Representative examples were shown for the production of ROS in PGRN KO and WT bone marrow-derived macrophages (n = 5 per group) at 2 hours after stimulation with heat-killed *C*. *albicans* yeast (at a multiplicity of infection of 1:20). (B) Representative examples were shown for the production of ROS in PGRN KO and WT neutrophils (n = 5 per group) at 2 hours after stimulation with heat-killed *C*. *albicans* yeast (at a multiplicity of infection of 1:20).
(TIF)

S5 Fig PGRN decreased Dectin-2 expression on the surface of macrophages and neutrophils. PGRN-deficient BMDM (n = 5) and neutrophils (n = 5) were preincubated with recombinant murine PGRN (100 ng/ml) for 2 hours and then challenged with heat-killed *C*. *albicans* yeast at a multiplicity infection of 1:10 for 30 min, and Dectin-2 expression level on the surface of BMDM and neutrophils was analyzed by flow cytometry. The Mann–Whitney *U* test was used to analyze the difference between groups denoted by horizontal lines, and *p* values were shown. All data were pooled from three independent experiments. MFI = mean fluorescence intensity.
(TIF)

S6 Fig PGRN deletion induced antifungal gene signatures in macrophages and neutrophils. (A) Volcano plots showed differentially-expressed genes (DEGs) between WT and PGRN-deficient BMDM (n = 3) upon live *C*. *albicans* stimulation (at a multiplicity of infection of 1:10) for 6 hours. The red dots represented up-regulated DEGs, whereas the cyan dots represented the down-regulated DEGs. (B) Gene set enrichment analysis (GSEA) identified “defense response to other organism” as the top 1 Gene Ontology (GO) terms with the highest normalized enrichment score in PGRN-deficient BMDM compared to WT cells. (C) GSEA of PGRN–related “defense response to other organism” gene signatures in BMDM. (D) Unsupervised hierarchical clustering heatmap of the highest DEGs by RNA-sequencing between WT and PGRN-deficient BMDM (n = 3 per group) upon live *C*. *albicans* stimulation (at a multiplicity of infection of 1:10) for 6 hours. (E) Volcano plots showed DEGs between WT and PGRN-deficient neutrophils (n = 3) upon live *C*. *albicans* stimulation (at a multiplicity of infection of 1:10) for 6 hours. (F) Unsupervised hierarchical clustering heatmap of the highest DEGs between WT and PGRN-deficient neutrophils (n = 3 per group) upon live *C*. *albicans* stimulation (at a multiplicity of infection of 1:10) for 6 hours. (G) GSEA of PGRN–related “defense response to fungus” gene signatures in neutrophils. (H) Gene set enrichment analysis identified “defense response to fungus” as the top 3 GO terms with the highest normalized enrichment score in PGRN-deficient neutrophils compared to WT cells.
(TIF)

S1 Data Source file. (XLSX)
