# Supplementary figures and images for: Progranulin aggravates lethal Candida albicans sepsis by regulating inflammatory response and antifungal immunity
Source: PLoS Pathog. 2022 Sep 19;18(9):e1010873. doi: 10.1371/journal.ppat.1010873 (PMC9521894; doi:10.1371/journal.ppat.1010873)

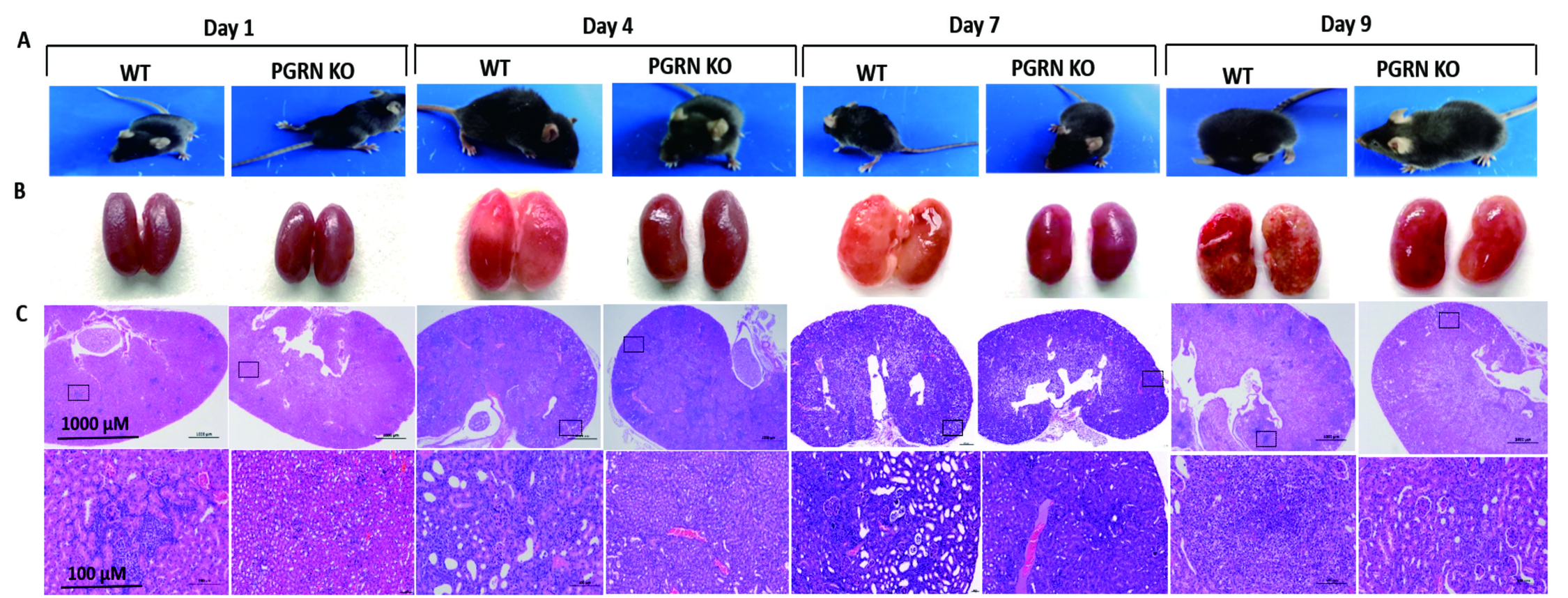

Supplement: S1 Fig — PGRN knockout (KO) and wild type (WT) mice were infected intravenously with 4 × 10^5^ colony forming units (CFU) of C. albicans. (A) Representative clinical appearance of WT and PGRN KO mice (n = 5 per group) after invasive C.albicans infection. (B) Anatomic pathology of kidneys in WT and PGRN KO mice (n = 5 per group) after invasive C.albicans infection. (C) Representative haematoxylin and eosin (HE) staining of kidney sections from WT and PGRN KO (n = 5 per group) mice after invasive C.albicans infection. Bottom panels are high magnification. Scale bars are 100 μM. (TIF) [file ppat.1010873.s001.tif]

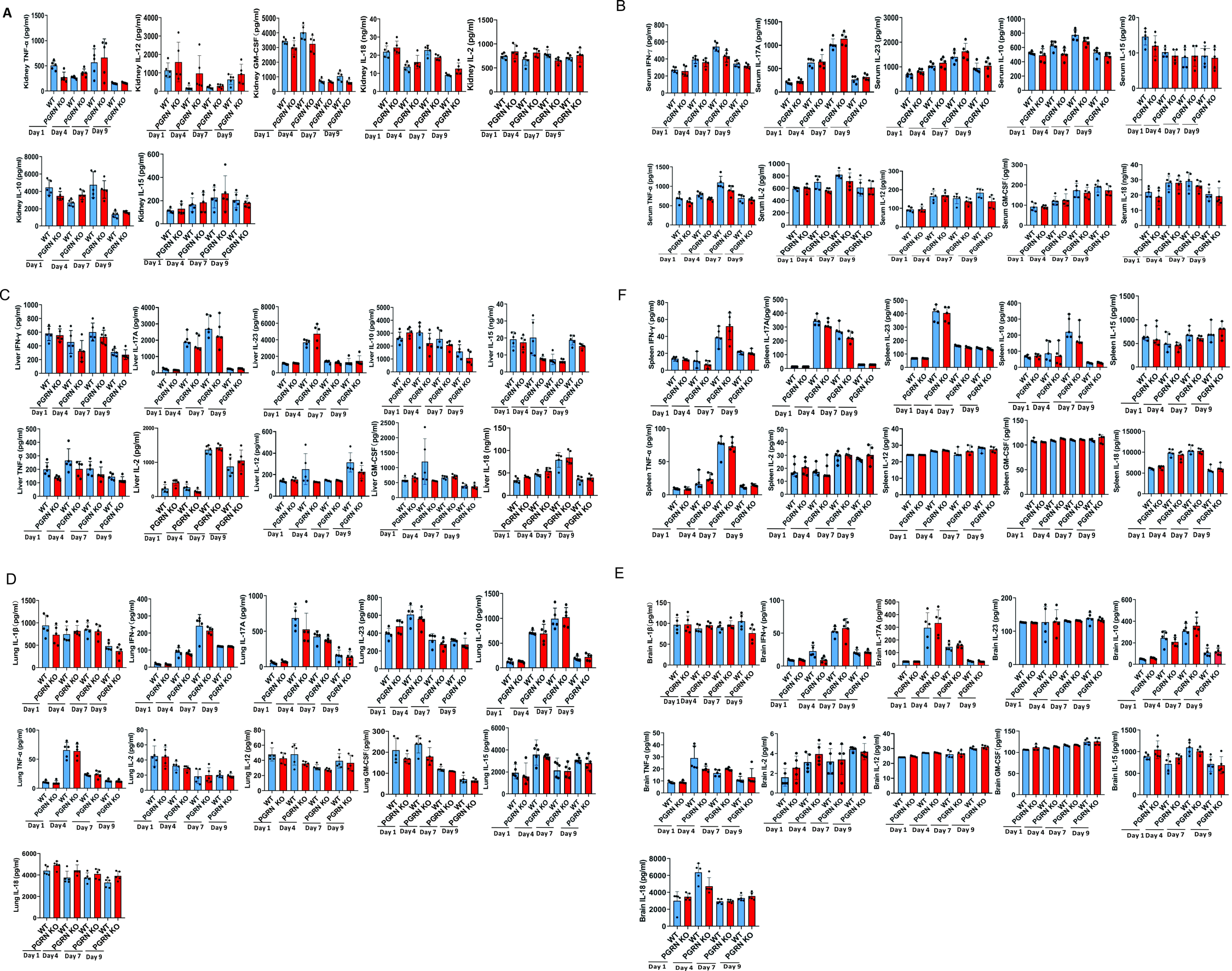

Supplement: S2 Fig — PGRN knockout (KO) and wild type (WT) mice were infected intravenously with 4 × 10^5^ colony forming units (CFU) of C. albicans. (A) Cytokines and chemokines in the kidneys (n = 5) were quantified by ELISA at the indicated times after C. albicans infection. (B) Cytokines and chemokines in the sera (n = 5) were quantified by ELISA at the indicated times after C. albicans infection. (C) Cytokines and chemokines in the livers (n = 5) were quantified by ELISA at the indicated times after C. albicans infection. (D) Cytokines and chemokines in the lungs (n = 5) were quantified by ELISA at the indicated times after C. albicans infection. (E) Cytokines and chemokines in the brains (n = 5) were quantified by ELISA at the indicated times after C. albicans infection. (F) Cytokines and chemokines in the spleens were quantified by ELISA at the indicated times after C. albicans infection. (TIF) [file ppat.1010873.s002.tif]

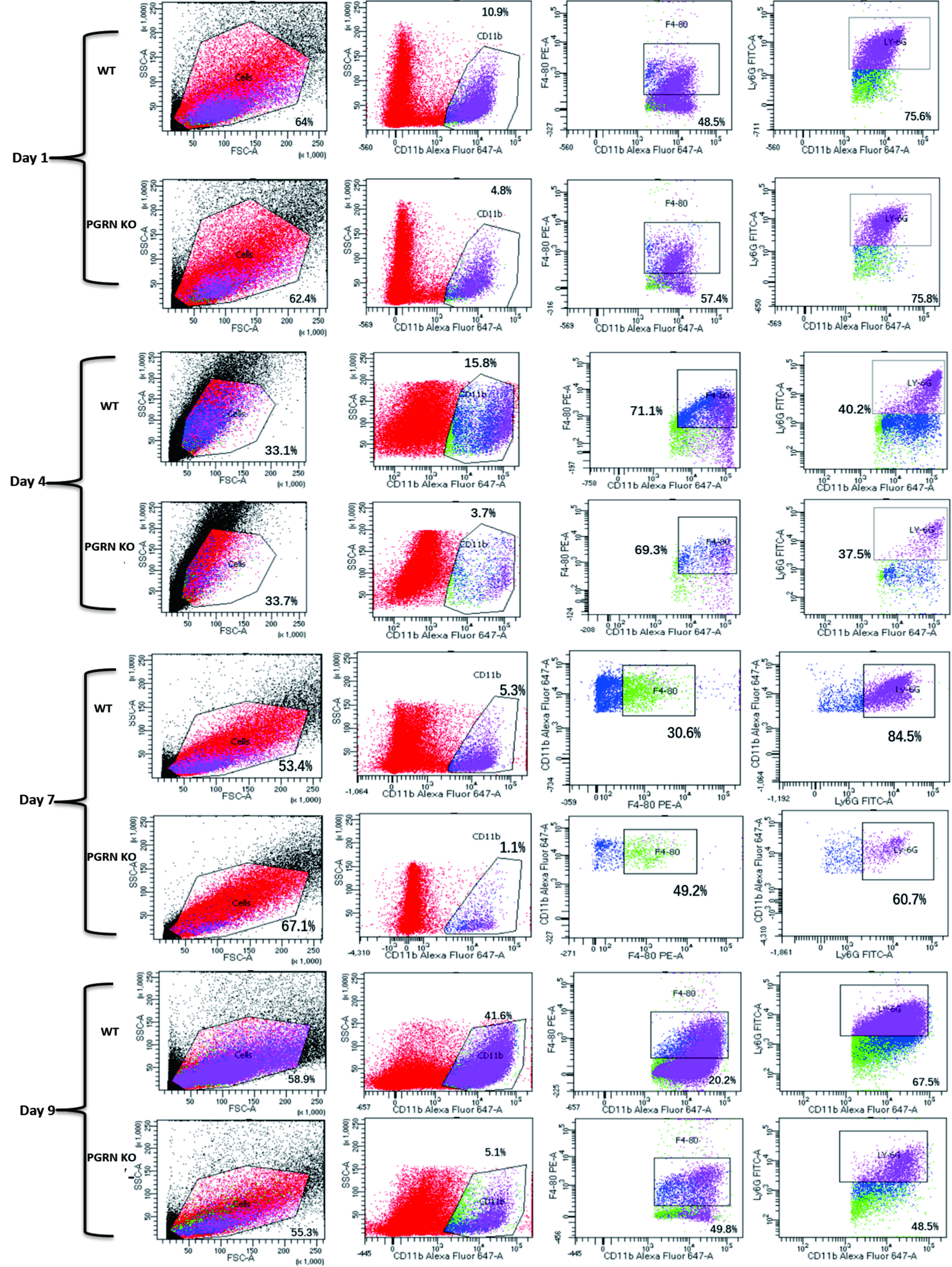

Supplement: S3 Fig — Flow cytometry gating scheme determining the percentage of mouse macrophages and neutrophils in the kidneys from PGRN KO and WT mice after C. albicans infection. Representative FACS plots and percentages of macrophages (CD11b+ F4/80+) and neutrophils (CD11b+ Ly-6G+) from five independent experiments were shown. (TIF) [file ppat.1010873.s003.tif]

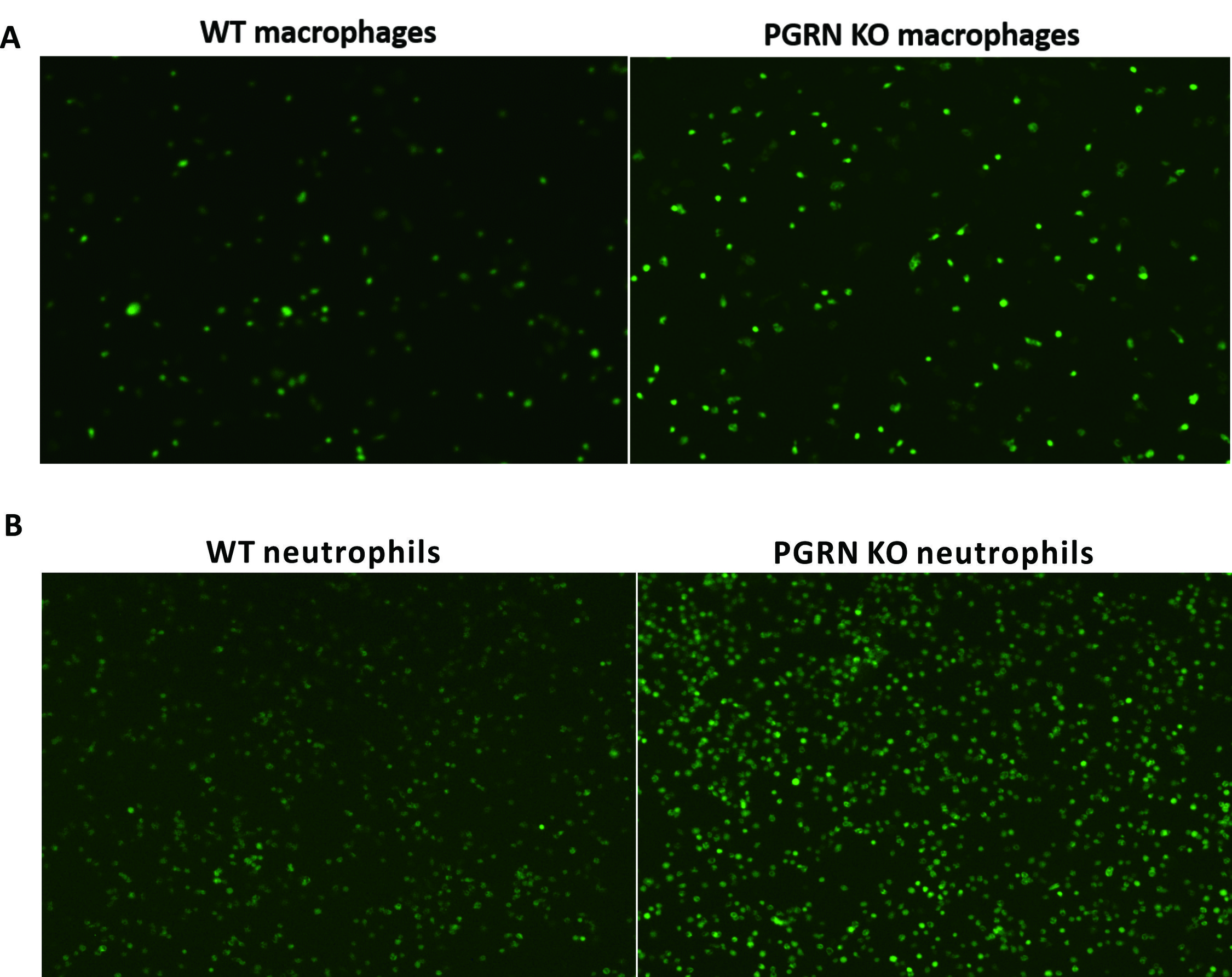

Supplement: S4 Fig — (A) Representative examples were shown for the production of ROS in PGRN KO and WT bone marrow-derived macrophages (n = 5 per group) at 2 hours after stimulation with heat-killed C. albicans yeast (at a multiplicity of infection of 1:20). (B) Representative examples were shown for the production of ROS in PGRN KO and WT neutrophils (n = 5 per group) at 2 hours after stimulation with heat-killed C. albicans yeast (at a multiplicity of infection of 1:20). (TIF) [file ppat.1010873.s004.tif]

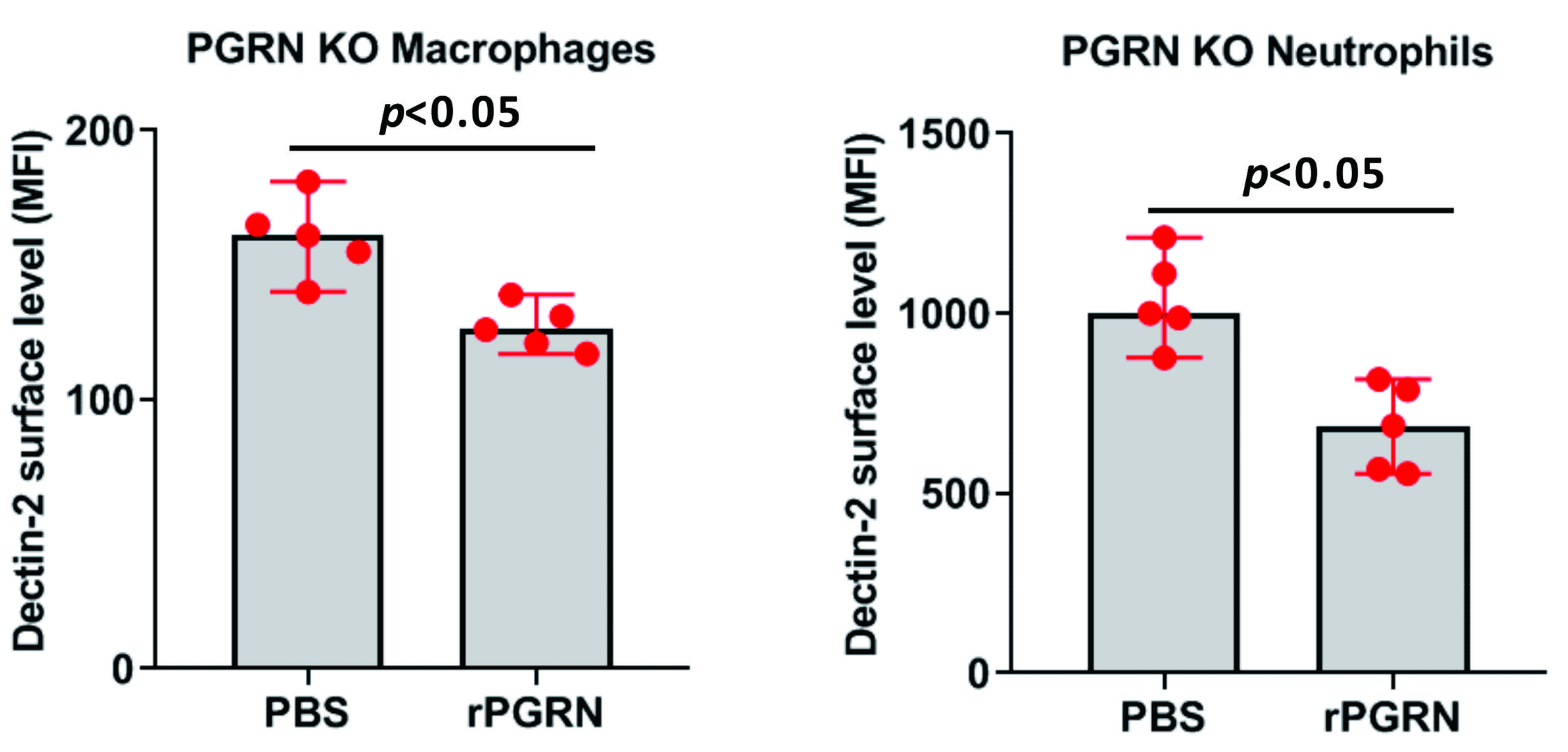

Supplement: S5 Fig — PGRN-deficient BMDM (n = 5) and neutrophils (n = 5) were preincubated with recombinant murine PGRN (100 ng/ml) for 2 hours and then challenged with heat-killed C. albicans yeast at a multiplicity infection of 1:10 for 30 min, and Dectin-2 expression level on the surface of BMDM and neutrophils was analyzed by flow cytometry. The Mann–Whitney U test was used to analyze the difference between groups denoted by horizontal lines, and p values were shown. All data were pooled from three independent experiments. MFI = mean fluorescence intensity. (TIF) [file ppat.1010873.s005.tif]

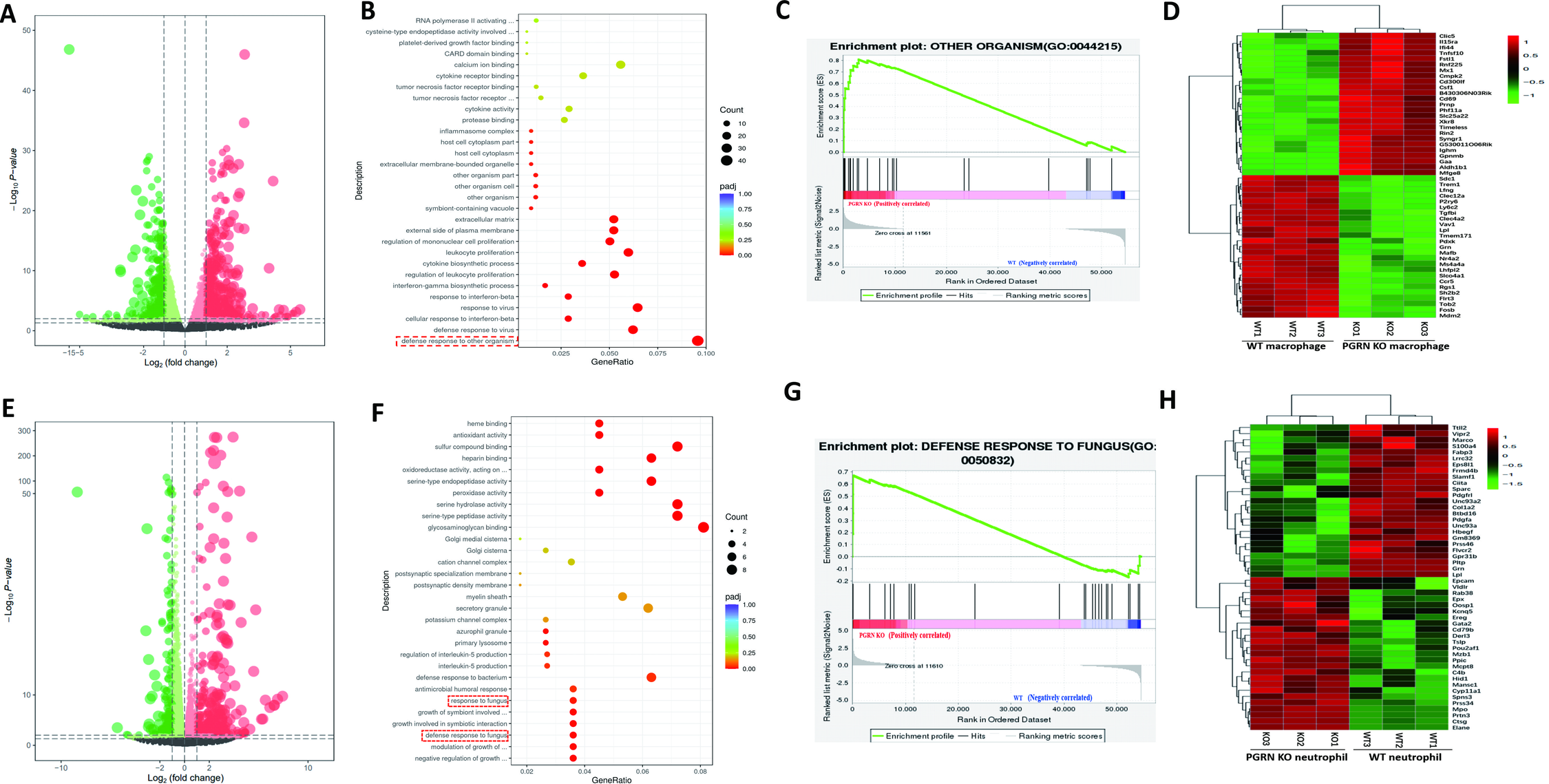

Supplement: S6 Fig — (A) Volcano plots showed differentially-expressed genes (DEGs) between WT and PGRN-deficient BMDM (n = 3) upon live C. albicans stimulation (at a multiplicity of infection of 1:10) for 6 hours. The red dots represented up-regulated DEGs, whereas the cyan dots represented the down-regulated DEGs. (B) Gene set enrichment analysis (GSEA) identified “defense response to other organism” as the top 1 Gene Ontology (GO) terms with the highest normalized enrichment score in PGRN-deficient BMDM compared to WT cells. (C) GSEA of PGRN–related “defense response to other organism” gene signatures in BMDM. (D) Unsupervised hierarchical clustering heatmap of the highest DEGs by RNA-sequencing between WT and PGRN-deficient BMDM (n = 3 per group) upon live C. albicans stimulation (at a multiplicity of infection of 1:10) for 6 hours. (E) Volcano plots showed DEGs between WT and PGRN-deficient neutrophils (n = 3) upon live C. albicans stimulation (at a multiplicity of infection of 1:10) for 6 hours. (F) Unsupervised hierarchical clustering heatmap of the highest DEGs between WT and PGRN-deficient neutrophils (n = 3 per group) upon live C. albicans stimulation (at a multiplicity of infection of 1:10) for 6 hours. (G) GSEA of PGRN–related “defense response to fungus” gene signatures in neutrophils. (H) Gene set enrichment analysis identified “defense response to fungus” as the top 3 GO terms with the highest normalized enrichment score in PGRN-deficient neutrophils compared to WT cells. (TIF) [file ppat.1010873.s006.tif]
